# Spatiotemporal Computational Meta‐Imager for Broadband Dual‐Layer Optical Convolution

**DOI:** 10.1002/advs.78340

**Published:** 2026-10-18

**Authors:** Mengguang Wang, Chenglong Zheng, Huaping Zang, Changwei Zhang, Huai Xia, Chang Wang, Zhenrong Zheng

**Affiliations:** ^1^ College of Optical Science and Engineering Zhejiang University Hangzhou China; ^2^ Key Laboratory of Material Physics, Ministry of Education, School of Physics Zhengzhou University Zhengzhou China; ^3^ Zhangjiang Laboratory Shanghai China

## Abstract

Optical neural networks face a tradeoff between device footprint and computational expressivity: deep systems are bulky, whereas single‐layer hardware lacks intrinsic depth and native negative‐weight support. We experimentally demonstrate a single‐layer metalens imager with “spatiotemporal intrinsic depth” for equivalent broadband dual‐layer convolution. A polarization‐multiplexed metasurface provides fixed Bessel‐vortex channels (*l* = 0 to 3) as the immutable first convolution layer, where the (*l* = 0) channel implements a hardware‐native residual connection. A metasurface‐generated 3 × 3 optical vortex matrix forms the second functional layer; time‐sequential liquid‐crystal amplitude modulation delivers tunable positive/negative weights. This temporal encoding solves the optical sign problem and converts fabrication imperfections into compensable biases via in‐situ training. Experiments verify rotational robustness for fashion‐item classification, raise full‐color CIFAR‐10 accuracy from 57.5 % to 86.6 %, and reach 99.3 % on the SIPaKMeD medical dataset, offloading 99.7 % of convolutional multiply‐accumulate operations to optics. By removing volumetric diffractive layers, this work enables low‐latency hybrid optoelectronic edge intelligent vision.

## Graphical abstract

A single‐layer polarization‐multiplexed meta‐imager achieves factorized dual‐layer broadband convolution via spatiotemporal kernel decomposition. Fixed Bessel‐vortex bases and time‐sequential liquid‐crystal modulation deliver tunable signed weights and in‐situ fabrication‐error compensation. Experiments verify improved classification performance across natural‐image and medical datasets while offloading nearly all convolutional computations to optics.

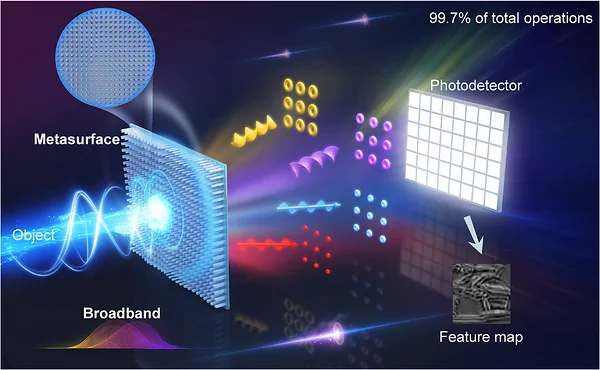

## 1 Introduction

The rapid evolution of deep neural networks has revolutionized machine vision [1], enabling unprecedented performance in image classification [2], object detection, and semantic segmentation [3, 4]. However, this progress has been accompanied by an exponential growth in model complexity and computational demand. State‐of‐the‐art vision architectures routinely require billions of multiply–accumulate operations per inference, translating to substantial energy consumption and latency that are incompatible with edge deployment scenarios [5]. The challenge is particularly acute in autonomous systems—ranging from unmanned aerial vehicles to wearable medical devices—where the imaging and processing pipeline must simultaneously satisfy stringent constraints on size, weight, power consumption, and real‐time responsiveness. These competing requirements expose a fundamental tension in conventional electronic processing: the von Neumann bottleneck between image acquisition and computation imposes irreducible data movement costs that scale poorly with increasing sensor resolution and network depth. Consequently, there is a pressing need for new computational paradigms that can shift the burden of feature extraction away from digital post‐processing and into the physical layer of image formation.

Optics has long been recognized as a promising avenue for alleviating the computational burden of convolution operations, which constitute the dominant fraction of arithmetic workload in modern vision networks [6, 7, 8, 9, 10]. The Fourier‐transform property of free‐space propagation enables massively parallel, passive execution of spatial filtering at the speed of light [11, 12]. Early optical correlators and more recent diffractive deep neural networks have demonstrated the viability of this approach, using cascaded phase masks to approximate multi‐layer perceptrons in the optical domain [13, 14]. Yet, these implementations typically rely on coherent illumination and require re‐imaging of digital data onto spatial light modulators, rendering them better suited as back‐end processors than as integrated front‐end imagers [15, 16]. Furthermore, the volumetric stacking inherent to multi‐layer diffractive networks directly contradicts the miniaturization imperative for edge deployment [13, 17, 18, 19, 20]. A transformative opportunity therefore lies in compressing the expressive capacity of multiple optical layers into a single, ultra‐compact optical element that operates directly on the incident scene—effectively embedding depth into the physical structure of the imager itself.

Metasurfaces [21, 22, 23, 24]—planar arrays of subwavelength scatterers that impart tailored phase, amplitude, and polarization modulation to incident light—offer a compelling platform for realizing front‐end optical computation in a form factor compatible with chip‐scale integration [25, 26, 27, 28, 29, 30, 31, 32, 33]. By engineering the meta‐atom response as a function of angle, wavelength, or polarization, researchers have demonstrated compact analogues of mathematical operations such as spatial differentiation and edge detection without the need for bulky 4F Fourier optics [34, 35, 36, 37, 38]. Multiplexing strategies have further enabled multi‐channel operation and, through optoelectronic subtraction, the realization of negative‐valued kernels under incoherent illumination [39, 40]. Despite these advances, existing metasurface processors remain constrained in their effective architectural depth. They are typically limited to a single fixed convolution layer with positively valued weights, or to multichannel kernels whose stride equals their size—a restriction that precludes the densely overlapping receptive fields and hierarchical feature extraction that define modern convolutional neural networks [41, 42, 43, 44, 45, 46, 47]. Bridging this gap requires an optical element that can simultaneously emulate multiple convolution layers, support arbitrary signed weights, and maintain operation across the broadband spectrum required for color imaging—all while offering a mechanism to compensate for the inevitable imperfections of nanoscale fabrication.

In this work, we introduce and experimentally validate a single metasurface aperture that computationally implements a dual‐layer convolutional kernel—a design we refer to as computational depth in the context of factorized kernel reconfigurability (Figure 1a). Unlike conventional diffractive networks that cascade discrete physical planes, our design leverages polarization‐division multiplexing to embed an immutable first convolutional layer directly into the transmission matrix of a single flat optic, while reserving a dedicated channel for hardware‐native residual connectivity. A second, reconfigurable convolutional layer is realized by an optical vortex matrix whose weight amplitudes and signs are dynamically controlled via time‐sequential liquid‐crystal modulation. This temporal encoding strategy not only circumvents the sign limitations of intensity‐based optics but uniquely enables in situ training, allowing the system to self‐calibrate against fabrication deviations (Figure 1b). We further demonstrate that the broadband dispersion of the Bessel–vortex basis sustains the convolution fidelity across the infrared spectral range, extending the operational scope to full‐color imagery (Figure 1c). The proposed architecture effectively folds two factorized convolution stages into a single optical pass, establishing a pathway toward ultra‐compact, task‐specific intelligent imagers that offload the vast majority of convolutional arithmetic to the front‐end optics. We substantiate these claims through a series of experiments spanning rotational robustness, color‐image classification, and high‐resolution medical imaging (Note S1 and Figure S1), illustrating the transformative potential of physically embedded computation for next‐generation edge vision systems (Figure 1d–f).

**FIGURE 1:**
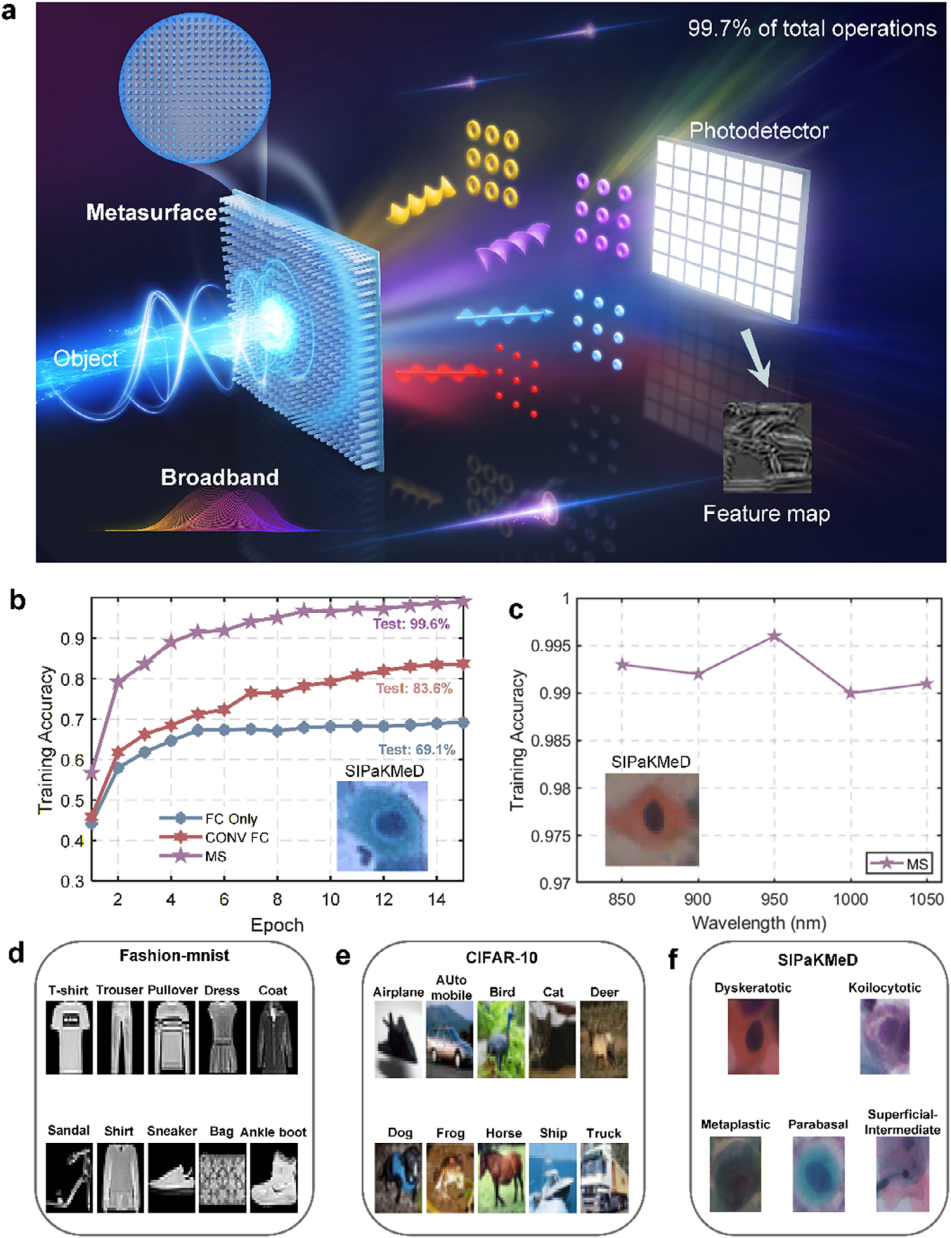
Schematic and performance overview of the spatiotemporal intrinsic depth meta‐imager. (a) Conceptual illustration of the single‐layer metasurface processor. The meta‐imager replaces digital convolution operations by performing multichannel signal processing directly in the optical domain. A polarization‐multiplexed bilayer meta‐optic encoding strategy—comprising a fixed first layer of Bessel‐vortex channels and a reconfigurable second layer of vortex matrices—enables broadband optical convolution under incoherent illumination. Positive and negative kernel weights are distinguished and recorded as feature maps by a polarization‐sensitive photodetector array, where each pixel integrates an oriented grating for on‐chip polarization sorting. (b) Comparative classification accuracy curves for three processing architectures: a fully connected digital network (FC Only), a single‐layer digital convolutional network (CONV FC), and the proposed meta‐imager front‐end followed by a lightweight digital classifier. (c) Measured classification accuracy of the meta‐imager across the broadband near‐infrared spectrum, demonstrating wavelength‐robust operation enabled by the engineered dispersion of the Bessel‐vortex basis (Table S6). (d) Representative sample images from the Fashion‐MNIST dataset used for rotation‐robustness validation. (e) Sample images from the full‐color CIFAR‐10 dataset. (f) Sample images from the high‐resolution SIPaKMeD medical cytology dataset employed for online training and error‐correction validation. Scale bars in (d–f) correspond to 28, 32, and 128 pixels, respectively. Representative test samples are sourced from the public Fashion‐MNIST, CIFAR‐10, and SIPaKMeD benchmark datasets.

## 2 Results and Discussion

### 2.1 Architecture of the Spatiotemporal Intrinsic Depth Meta‐Imager

In a standard digital convolutional neural network, feature extraction is performed by sliding an *N* × *N* trainable kernel *K* across the input image *I*_in _ and computing the local dot product at each spatial location. As illustrated in Figure 2a, the output value at pixel (*i*, *j*) is given by.

(1) $$I_{\mathrm{out}\;}\left(i,j\right)=\sum_{p=1}^{N}\sum_{q=1}^{N}K\left(p,q\right)\cdot I_{\mathrm{in}\;}\left(i+p-1,j+q-1\right)$$

**FIGURE 2:**
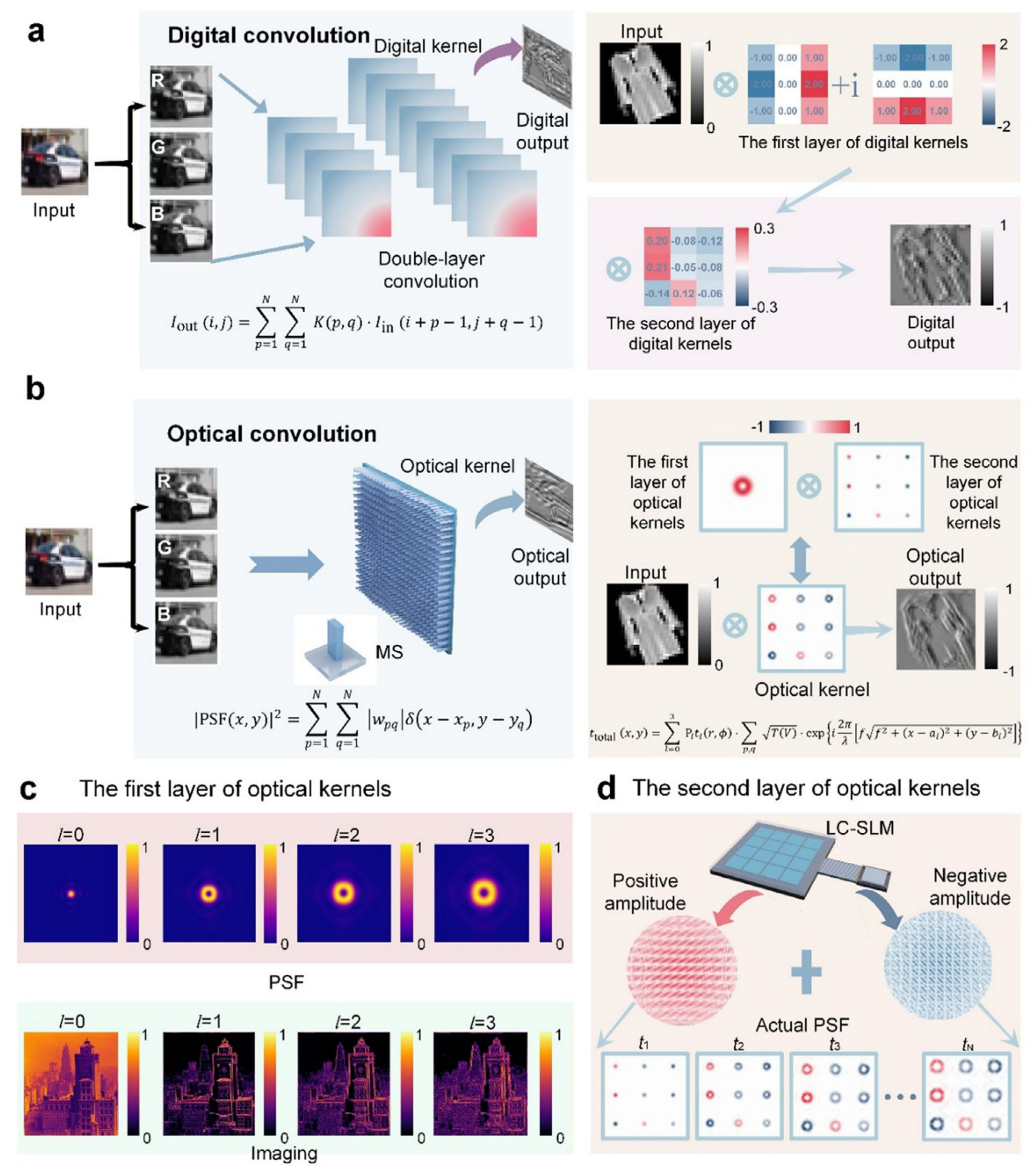
Principle of dual‐layer optical convolution enabled by a single‐layer metasurface. (a) Schematic of digital convolution. An N × N trainable kernel (illustrated here for N = 3) slides across the input image with a defined stride, performing pixel‐wise dot products to generate a feature map. This operation constitutes the dominant computational burden in conventional convolutional neural networks. (b) Optical implementation of two‐layer convolution in a single metasurface. Fixed first‐layer kernels are provided by four polarization‐multiplexed Bessel‐vortex channels (*l* = 0,1,2,3) with intensity profiles. A reconfigurable second layer is formed by a 3 × 3 focal‐spot array with liquid‐crystal amplitude control. The cascaded operation—first‐layer Bessel filtering followed by second‐layer weighted shifting—is executed entirely in the optical domain. Signed weights are realized via temporal differential detection. (c) First convolutional layer: polarization‐multiplexed Bessel‐vortex basis. Four orthogonal polarization channels independently generate Bessel‐vortex modes with topological charges *l* = 0,1,2,3. The upper panel displays the computed intensity profiles of each vortex beam at the focal plane, showing characteristic ring‐like distributions with central maxima (*l* = 0) or central nulls (*l* = 1,2,3) of increasing radius. The lower panel illustrates the effect of each channel on a representative input image: the l = 0 channel preserves global luminance (low‐pass filtering and hardware‐native residual connection), while *l* = 1,2,3 perform increasing orders of spatial differentiation, extracting edges, textures, and curvature features. These four operations are fixed by metasurface design and constitute the immutable first convolutional layer. (d) Second convolutional layer: reconfigurable 3 × 3 optical vortex matrix. The metasurface further encodes a 3 × 3 array of focal spots, each carrying an independent vortex phase. The panel shows the target discrete PSF with nine spatially separated foci arranged on a regular grid. The relative brightness of each focus is dynamically controlled by a liquid‐crystal amplitude modulator, which applies spatially varying transmission coefficients *T*(*V*) in response to applied voltages. The panel depicts the reconfigurable kernel weights: through temporal differential encoding, both positive (red) and negative (blue) weight values are physically realized, enabling fully signed, programmable convolution kernels. The combination of the fixed first‐layer Bessel priors (c) and the tunable second‐layer vortex matrix (d) folds two complete convolutional layers into a single sub‐millimeter‐thick metasurface.

This operation is repeated across all positions, producing a feature map that encodes local correlations at a scale determined by the kernel size *N*. The computational cost scales linearly with the number of pixels and quadratically with *N*, quickly becoming the dominant arithmetic burden in deep vision pipelines. To alleviate this load, one may seek to transfer the convolution operation into the optical domain, where free‐space propagation inherently performs spatial averaging at the speed of light.

The bridge between digital and optical convolution is established by recognizing that an incoherent imaging system forms an image through the convolution of the object intensity with the modulus squared of its coherent point spread function (PSF). If the PSF of an optical front end can be engineered to mimic the structure of a digital kernel, then the detected image will directly represent the convolved feature map. Our approach accomplishes this by designing a metasurface that generates a discrete *N* × *N* array of diffraction‐limited focal spots in the image plane, as depicted in Figure 2b. The intensity distribution of this engineered PSF can be written as:

(2) $${\left|PSF\left(x,y\right)\right|}^{2}=\sum_{p=1}^{N}\sum_{q=1}^{N}\left|w_{pq}\right|\delta \left(x-x_{p},y-y_{q}\right)$$

where (*x_p_*, *y_q_*) are the coordinates of the focal spots arranged on a regular grid, and |*w_pq_* | quantifies the relative brightness of each spot. Substituting Equation 2 into the incoherent imaging equation yields an output that is precisely the weighted sum of shifted copies of the input scene, thereby achieving a mathematical equivalence to the digital convolution in Equation 1. The sign of each weight, which is inherently positive in an intensity‐only measurement, is later resolved through a temporal differential detection scheme described below.

### 2.2 The First Convolutional Layer: Polarization‐Multiplexed Bessel‐Vortex Priors

With the optical convolution framework established, we now detail the physical realization of a two‐layer convolutional architecture within a single metasurface. The first layer is implemented through four independent polarization channels, each producing a distinct Bessel‐vortex mode. In polar coordinates (r,ϕ) at the metasurface plane, the complex transmission coefficient of the *l*‐th channel is given by

(3) $$t_{l}\left(r,\phi \right)=J_{l}\;\left(\alpha r\right)e^{il\phi }e^{-ik_{0}\frac{r^{2}}{2f}}$$

where *l* ∈ {0, 1, 2, 3} is the topological charge, *J_l_* is the *l*‐th order Bessel function of the first kind, *k*_0_ = 2π/*λ* is the free‐space wavenumber, *f* is the focal length of the metalens, and *α* is a scaling constant that sets the radial extent of the Bessel mode. The quadratic phase term $\;e^{-ik_{0}\frac{r^{2}}{2f}}$ imparts the focusing power of a conventional lens, collapsing the Bessel distribution into a sharply defined ring‐like focus at the back focal plane (Note S2 and Figure S2).

Crucially, each topological charge l corresponds to a specific spatial filtering operation (Figure 2c). The *l* = 0 channel produces a central bright maximum and functions as a low‐pass filter that preserves global luminance—this channel serves as a hardware‐native residual connection, providing a direct pathway for unprocessed image content to bypass the convolutional layers. The *l* = 1 channel generates a doughnut‐shaped focus with a central null, approximating a first‐order spatial derivative that extracts edges and gradients. The *l* = 2 and *l* = 3 channels impose higher‐order spatial differentiation, capturing curvature and fine textural variations. These four operations are fixed by the metasurface design and are applied in parallel to the incoming scene, irrespective of the downstream task. They constitute an immutable first convolutional layer that injects well‐understood physical priors into the processing pipeline, significantly reducing the learning burden on any subsequent digital classifier.

### 2.3 The Second Convolutional Layer and the Fusion of Two Depths

The second convolutional layer is realized through a metasurface‐generated 3 × 3 array of optical vortices, each of which acts as a discrete, reconfigurable weight element (Note S3 and Figure S3). Unlike the fixed first layer, the intensities of these nine focal spots are dynamically controlled by a liquid‐crystal (LC) amplitude modulator placed in the optical path. The LC layer is an independently addressable pixelated spatial light modulator, in which each pixel's transmission coefficient *T*(*V*(*x*, *y*))∈[0, 1] is controlled by an applied voltage. The modulator is programmed with a spatially varying transmission map that independently sets the relative intensity of each focal spot in the 3 × 3 vortex array. To incorporate both positive and negative weights—a necessity for arbitrary convolutional kernels—we employ a temporal differential encoding strategy. Each target weight *w_pq_* is decomposed as $w_{pq}=w_{pq}^{+}-w_{pq}^{-}$, with $w_{pq}^{\pm }\geq 0$,. In two successive acquisition frames, the LC modulator applies transmission factors corresponding to the positive and negative matrices, respectively, and the detector records two intensity distributions *I* ^+^ and *I* ^−^. The final feature map is obtained by digital subtraction *I*_out _ = *I* ^+^  − *I* ^−^, which not only yields signed kernel values but also suppresses common‐mode noise sources such as detector dark current and ambient background. The complex amplitude profile of the optical setup is described by the following model:

(4) $$\begin{array}{cc} & t_{\mathrm{total}\;}\;\left(x,y\right)=\sum_{l=0}^{3}P_{l}t_{l}\;\left(r,\phi \right)\cdot \sum_{p,q}\sqrt{T\left(V\left(x,y\right)\right)}\cdot \\ & \exp\left\{i\frac{2\pi }{\lambda }\left[f\sqrt{f^{2}+{\left(x-a_{i}\right)}^{2}+{\left(y-b_{i}\right)}^{2}}\right]\right\} \end{array}$$

P_*l*_ is the polarization projection operator for the *l*‐th channel, ensuring that the channels are orthogonally separated in the polarization domain. The parameters *a_i_* and *b_i_* quantify the lateral offsets of the *i*‐th focal spot relative to the optical axis.

The fusion of the two convolutional layers occurs seamlessly in the optical domain. The four polarization‐multiplexed Bessel‐vortex channels from the first layer propagate through the same physical aperture and are simultaneously weighted by the LC‐modulated second‐layer kernel. Because the polarization states are orthogonal, the channels do not interfere; they are instead separated at the detection plane by a polarization‐sensitive photodetector array, where each pixel integrates an oriented grating for on‐chip polarization sorting. The result is a set of four distinct feature maps, each corresponding to a different combination of first‐layer prior and second‐layer tunable weights. This architecture effectively folds two complete convolutional layers into a single sub‐millimeter‐thick metasurface, exploiting polarization and time as orthogonal multiplexing dimensions to embed depth without volumetric stacking.

Finally, the temporal reconfigurability afforded by the liquid‐crystal amplitude modulator endows the system with a defining practical advantage: in situ error correction through online training. Nanofabrication inevitably introduces static phase deviations into the metasurface, and experimental alignment invariably involves minute mechanical misregistration. In a conventional passive metasurface, such imperfections would permanently degrade the fidelity of the optical convolution and remain uncorrectable post‐fabrication. Here, however, the hybrid optoelectronic training loop—wherein digital loss gradients are backpropagated to update the liquid‐crystal transmission map—enables the system to autonomously learn compensatory amplitude weightings during a brief calibration phase. This process effectively transforms a static, error‐prone nanostructure into an actively trainable optical layer that can self‐calibrate against both manufacturing variability and experimental setup discrepancies. The ability to absorb and neutralize these otherwise performance‐limiting deviations is the hallmark of the online training paradigm. Combined with the broadband dispersion engineering that maintains kernel geometric fidelity across the spectrum, this self‐correcting capability underpins robust, full‐color convolutional processing that offloads up to 99.7% of the multiply–accumulate operations from the digital backend to the optical front end (Note S4 and Table S1).

The hybrid optoelectronic architecture of our meta‐imager enables a unique closed‐loop training paradigm in which the optical front end, the digital back end, and an amplitude‐prediction neural network (APNN) are jointly optimized to compensate for both task‐specific feature requirements and static fabrication deviations. The complete workflow is schematically illustrated in Figure 3.

**FIGURE 3:**
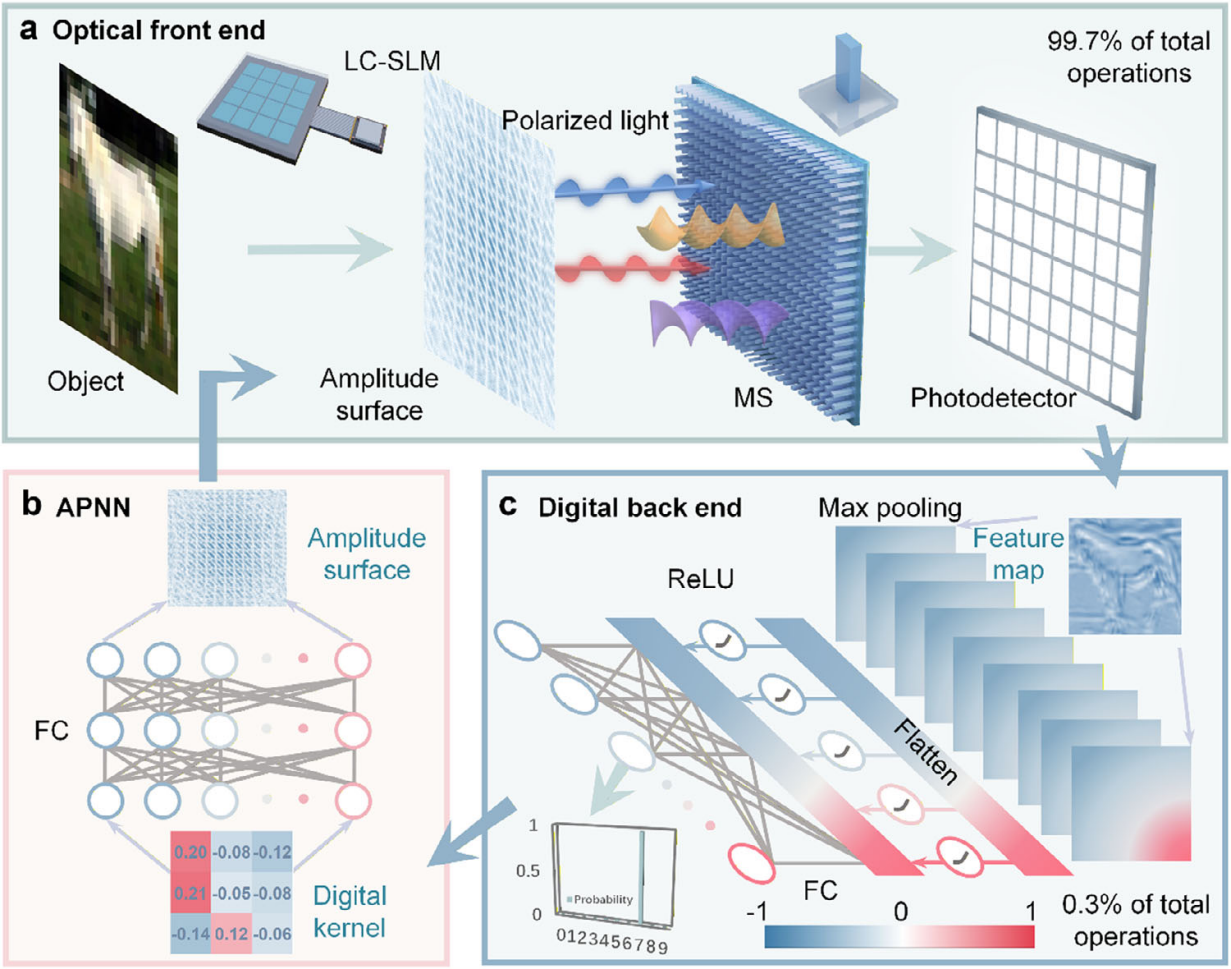
Closed‐loop online training architecture with spatiotemporal intrinsic depth. (a) Schematic of the self‐tunable metasurface front end. The device (Table S2) comprises an electrically addressable liquid‐crystal (LC) amplitude modulator cascaded with a polarization‐multiplexed dielectric metasurface (Notes S5 and S6 and Figures S4–S7). The metasurface encodes two distinct functional layers within a single physical aperture: a fixed first convolutional layer realized by four orthogonal polarization channels that generate Bessel‐vortex modes with topological charges *l* = 0,1,2,3, and a reconfigurable second convolutional layer formed by a 3 × 3 optical vortex matrix. Under broadband incoherent illumination, the LC layer applies a spatially varying transmission map  *T*(*V*(*x*, *y*)) to independently modulate the intensity of each focal spot in the vortex matrix. Temporal differential encoding across successive frames—alternating between positive and negative transmission matrices—enables the physical realization of signed kernel weights. The polarization‐sensitive photodetector captures the resulting four‐channel feature maps, which are subsequently routed to the digital back end. (b) APNN. The APNN is a lightweight encoder–decoder network that learns the nonlinear mapping from a target convolution kernel or desired PSF intensity distribution to the corresponding LC transmission matrix. The predicted transmission map is converted to voltage signals that drive the LC modulator. (c) Digital back end and closed‐loop training workflow. Optically convolved feature maps from the four polarization channels are captured by the photodetector and fed into a lightweight digital classifier consisting of a max‐pooling layer, a rectified linear unit (ReLU) activation, and a fully connected (FC) layer that outputs class probability histograms for image recognition. The cross‐entropy loss computed from the prediction is backpropagated through the digital layers and the optical PSF model to provide gradient signals for updating the LC transmission map. This gradient information is routed to the APNN as a refined target kernel specification, closing the feedback loop.

### 2.4 Digital Back End

The optical convolution layer produces four polarization‐multiplexed feature maps—one per Bessel‐vortex channel (*l* = 0,1,2,3)—each of which has been convolved with a reconfigurable 3 × 3 kernel encoded in the intensity distribution of the vortex‐matrix PSF. These feature maps are captured by a polarization‐sensitive CMOS sensor and serve as the input to a lightweight digital back end. The digital pipeline consists of a max‐pooling layer that downsamples the feature maps, followed by a rectified linear unit (ReLU) activation and a fully connected classification layer. The predicted class probabilities are obtained via a softmax function,

(5) y^=SoftmaxW·FlattenMaxPoolReLUIin⊛F−1ttotalx,y2+b

In the model, *W* corresponds to the modifiable weight matrix, *b* is the offset vector, and $F^{-1}$ denotes the inverse Fourier transform. The whole architecture not only outputs the prediction probability, but also back‐propagates the gradient information and optical path error to the amplitude‐prediction neural network (APNN).

### 2.5 An Amplitude‐Prediction Neural Network

The key innovation of our training scheme lies in treating the liquid‐crystal amplitude modulator not merely as a static weight provider, but as a continuously tunable parameter plane that can be updated in response to both digital loss gradients and measured optical imperfections. To accelerate this process, we employ a lightweight APNN that learns the nonlinear mapping from a target PSF intensity distribution to the required liquid‐crystal transmission matrix. The APNN is trained offline using 4000 pairs of synthetically generated target PSFs and their corresponding optimized LC transmission profiles, obtained via conventional gradient descent. Once trained, the APNN predicts an initial LC transmission map *T*(*V*(*x*, *y*)) for any desired kernel in approximately 0.8 ms, eliminating the need for time‐consuming iterative optimization at inference time (Note S7 and Figure S8).

During online training, the system operates in a closed loop. An input batch is passed through the optical layer, where the liquid‐crystal modulator applies the current transmission map *T*(*V*(*x*, *y*)). The resulting feature maps are digitized and processed by the digital back end to compute the cross‐entropy loss  $L=\mathrm{CrossEntropy}(\hat{y},y_{\mathrm{true}\;})$. The gradient of this loss with respect to the LC transmission values is then backpropagated through the digital layers and the optical PSF model, yielding an update direction for the amplitude plane. The transmission map is iteratively refined according to

(6) $$\begin{array}{c} T{\left(V\left(x,y\right)\right)}^{\left(t+1\right)}=T{\left(V\left(x,y\right)\right)}^{\left(t\right)}-\eta \cdot \frac{\partial \mathrm{CrossEntropy}\;\left(\hat{y},y_{\mathrm{true}\;}\right)}{\partial T\left(V\left(x,y\right)\right)}\; \end{array}$$

where *η* is the learning rate and the gradient encapsulates both the digital classification error and the physical forward model of the PSF. This update rule adjusts the relative brightness of the nine vortex spots, thereby tuning the second‐layer convolutional kernel while the first‐layer Bessel‐vortex priors remain fixed.

Figure 4a presents a photograph of the fabricated dielectric metasurface. The device consists of a circular aperture with a diameter of 2 mm patterned on a transparent fused silica substrate, with the nanostructured region visible as a faint iridescent area at the center of the chip. High‐resolution scanning electron microscopy (SEM) imaging of the fabricated metasurface (Figure 4b) reveals a well‐ordered square lattice of silicon nanopillars. The pillars exhibit smooth sidewalls and uniform dimensions, with minor critical dimension variations below ±8 nm across the entire aperture. The core functionality of the meta‐imager—the generation of four polarization‐multiplexed Bessel‐vortex channels, each further structured into a discrete 3 × 3 focal‐spot array—was validated through both numerical simulation and direct optical measurement. The Bessel‐vortex modes (*l* = 0,1,2,3) constitute the fixed first convolutional layer that imparts physically grounded edge and texture priors, while the intensity distribution within each 3 × 3 array forms the reconfigurable second convolutional layer via the weighted superposition of shifted sub‐images at the detection plane. Figure 4c displays the simulated intensity PSF at the back focal plane (*f* = 1.25 mm) under illumination centered at 950 nm. The PSF exhibits nine well‐defined focal spots arranged on a regular Cartesian grid with a pitch of 7 µm, corresponding to a digital stride of one pixel on the sensor array. The relative intensities of the spots, color‐coded by their polarization channel of origin, are engineered to match a predefined set of convolution kernel weights. Slight variations in spot ellipticity are attributable to off‐axis aberrations, which are partially compensated by the LC amplitude tuning during online calibration. Experimental PSF measurements (Figure 4d), acquired using a collimated broadband source and a polarization‐sensitive CMOS sensor (Note S8 and Figure S9), show excellent agreement with the simulated intensity distributions in Figure 4c, confirming the high fidelity of the fabricated metasurface (Note S9 and Figure S10).

**FIGURE 4:**
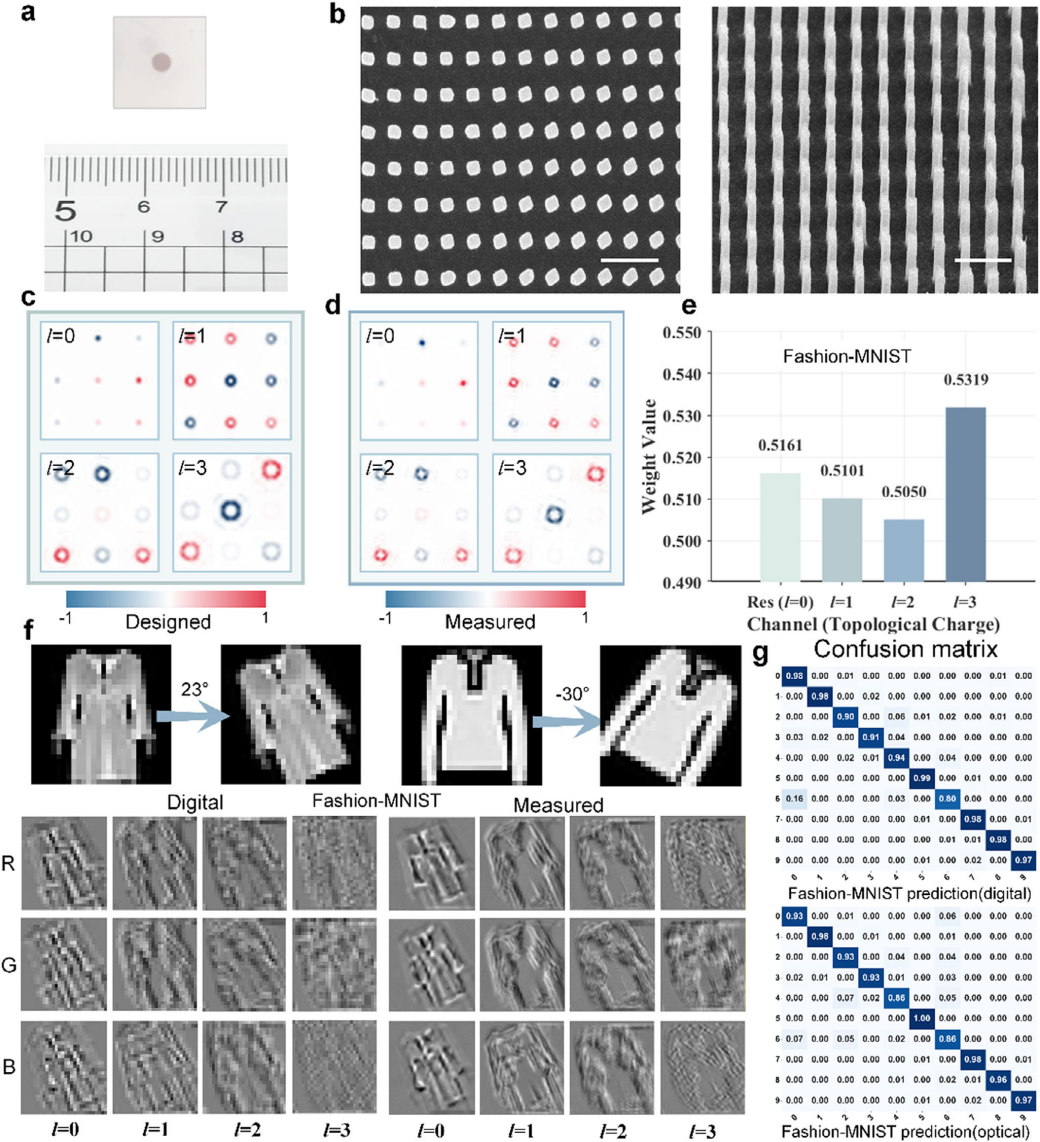
Experimental characterization and performance validation of the spatiotemporal intrinsic depth meta‐imager. (a) Photograph of the fabricated meta‐imager. The device integrates a polarization‐multiplexed dielectric metasurface with a wire‐bonded liquid‐crystal amplitude modulator, forming a compact, electrically addressable optical convolution front end. (b) Representative scanning electron microscopy (SEM) image of the metasurface nanostructure (scale bar: 1.04 µm). The metasurface comprises a square lattice of silicon nanopillars with subwavelength periodicity (520 nm) and varying in‐plane rotation angles to impart the required geometric and propagation phase profiles. For experimental convenience, the fabricated prototype comprises two adjacent metalens regions (to achieve superior polarization isolation), but the underlying design is a single metasurface aperture that computationally realizes the dual‐layer kernel. The conceptual device requires only one monolithic metasurface. (c) Simulated PSF of the designed metasurface under circularly polarized broadband illumination, showing the discrete 3 × 3 focal spot array with engineered relative intensities. The four polarization channels (*l* = 0,1,2,3) are superimposed in this composite intensity map. (d) Experimentally measured PSF at the back focal plane, captured using a polarization‐sensitive CMOS sensor. The focal spot positions and relative brightness closely match the simulated design in (c), confirming high‐fidelity nanofabrication and accurate phase retrieval. (e) Normalized channel weight distribution across the four polarization‐multiplexed Bessel‐vortex modes (*l* = 0,1,2,3). (f) Experimental validation of rotational robustness using the Fashion‐MNIST dataset. Upper panel: Representative input images subjected to random in‐plane rotations (0° to 180°). Left panel: Simulated feature maps after optical convolution, showing consistent structural fidelity across rotation angles. Right panel: Corresponding experimentally captured feature maps from the fabricated device, exhibiting strong agreement with simulation, and confirming the inherent rotation invariance conferred by the Bessel‐vortex basis (Tables S3 and S4). (g) Confusion matrices for digital and optical back‐end classification (Table S5).

Figure 4e displays the normalized channel weight distribution across the four polarization‐multiplexed Bessel‐vortex modes, revealing a balanced allocation in which the *l* = 0 residual channel retains sufficient optical power to preserve global image content while the higher‐order channels (*l* = 1,2,3) distribute the remaining intensity for multi‐scale spatial differentiation (Note S10 and Figure S11). This intrinsic weight partitioning acts as a physically embedded regularize that prevents any single feature type from dominating the representation, thereby enhancing feature diversity and directly contributing to the improved classification accuracy observed across all benchmark datasets. A distinguishing theoretical attribute of the Bessel‐vortex basis is its invariance to in‐plane image rotations: a rotation of the input scene merely imparts a global phase shift to the vortex modes, leaving the intensity distribution of the convolved feature maps unchanged (Table S7). To experimentally validate this property, we constructed a rotated test set from the Fashion‐MNIST dataset by applying random in‐plane rotations uniformly sampled from 0° to 180° to each grayscale image. Representative samples are shown in Figure 4f (Notes S11 and S12, Figures S12 and S13). We evaluated classification accuracy on this rotated test set using two configurations: the complete hybrid pipeline, in which the meta‐imager performs optical convolution followed by a lightweight digital classifier, and a digital‐only double‐layer convolutional network with matched kernel dimensions and channel count. The simulated double‐layer convolutional network convolution achieved an accuracy of 94.3%, while the experimental measurement from the fabricated meta‐imager reached 93.9%, demonstrating no appreciable degradation relative to the unrotated baseline. In comparison, the digital‐only single‐layer network attained only 89.4% accuracy. The confusion matrices comparing the hybrid and digital double‐layer models are presented in Figure 4g. The substantial accuracy gap—a 4.5 percentage point advantage for the optical front end—quantifies the expressive benefit of embedding two effective convolutional layers within the single metasurface and confirms that the Bessel‐vortex basis confers inherent robustness to rotational distortions without sacrificing classification fidelity.

Having characterized the static optical performance of the meta‐imager, we next evaluated its end‐to‐end classification capabilities on a full‐color natural image benchmark. We used the CIFAR‐10 dataset, comprising ten object categories (airplane, automobile, bird, cat, deer, dog, frog, horse, ship, and truck) with original 32 × 32 pixel spatial resolution and three RGB color channels. Representative input images are shown in Figure 5a. For each test sample, the meta‐imager processed all three‐color channels sequentially through the same physical aperture, with the liquid‐crystal amplitude modulator applying identical kernel weights across wavelengths—a capability enabled by the broadband dispersion engineering of the Bessel‐vortex basis described previously. The resulting optically convolved feature maps (Figure 5b) exhibit pronounced edge enhancement and texture extraction that are largely invariant to the intrinsic color variations within each object category (Notes S13 and S14, Figures S14 and S15). Figure 5c presents the confusion matrices comparing the classification performance of two configurations. A purely digital single‐layer convolutional network, constrained to the same kernel size and channel count, achieves a test accuracy of only 57.5% on CIFAR‐10 (Note S15 and Figure S16). When the digital network is expanded to two convolutional layers with an equivalent number of trainable parameters, the accuracy rises substantially to 87.5%, confirming that the additional depth provides critical expressive capacity for this task. Remarkably, the full hybrid pipeline—wherein the meta‐imager replaces the digital convolutional layers and a lightweight fully connected classifier processes the resulting optical feature maps—attains 86.6% accuracy. This value approaches the performance of the two‐layer digital network while employing only a single physical metasurface aperture. The negligible 0.9 percentage‐point gap relative to the digital two‐layer baseline, contrasted with the 29.1 percentage‐point gap relative to the digital single‐layer baseline, directly quantifies the expressive power of the meta‐imager's spatiotemporal intrinsic depth: the fixed Bessel‐vortex prior layer and the tunable vortex‐matrix layer together fold two effective convolutional layers into one flat optic, delivering the accuracy of a deeper network without the corresponding digital computational burden. That classification fidelity is preserved—and indeed slightly enhanced—while offloading the dominant fraction of arithmetic to the optical domain constitutes a compelling validation of the meta‐imager's practical utility.

**FIGURE 5:**
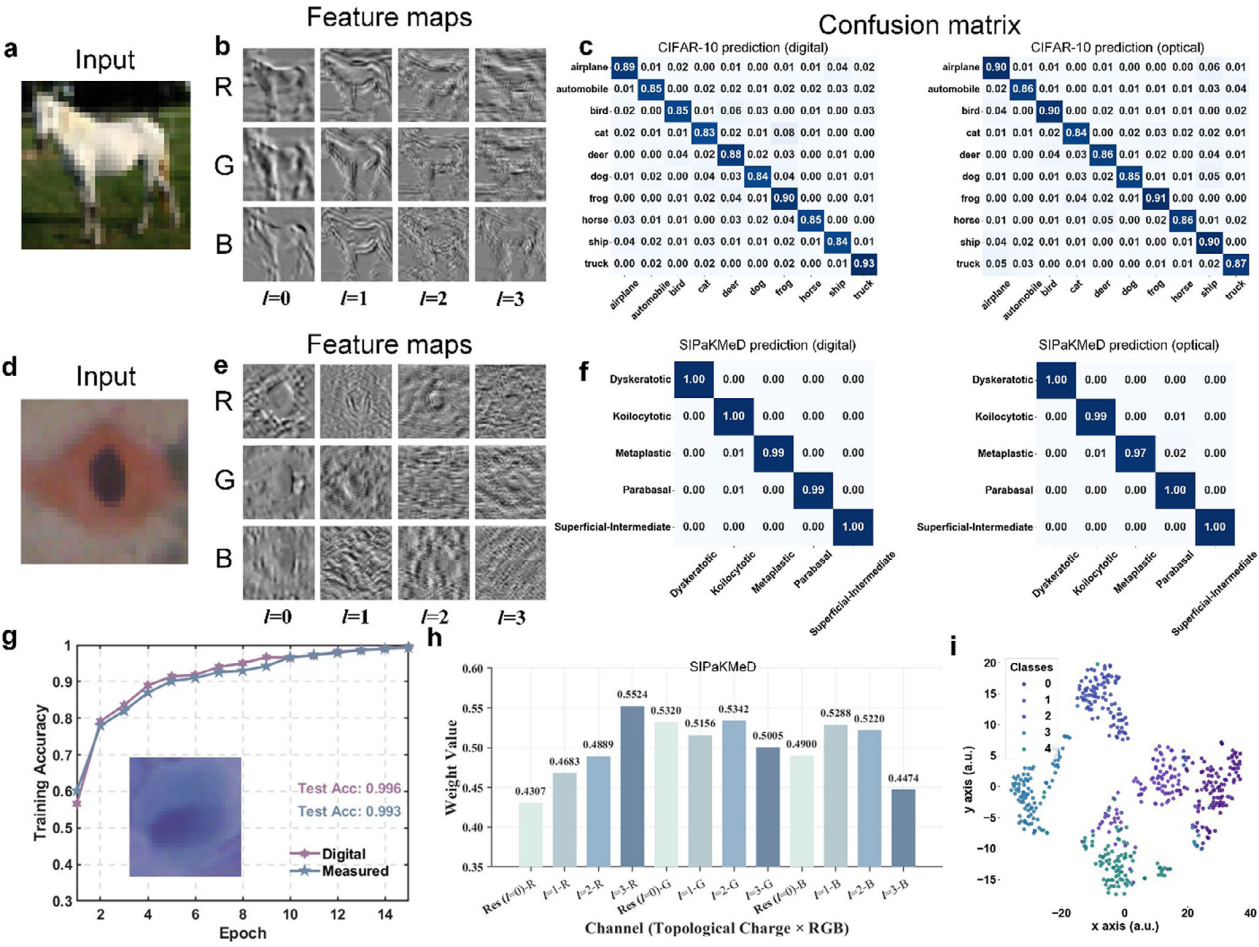
Experimental validation on full‐color and high‐resolution medical imaging benchmarks. (a) Representative input images from the CIFAR‐10 dataset. Scale bar: 32 pixels. (b) Corresponding optically convolved feature maps captured at the meta‐imager output plane. The four polarization channels (*l* = 0,1,2,3) are digitally combined into a single composite visualization; the edge‐enhanced structures and color‐invariant texture extraction are evident across diverse object classes. (c) Confusion matrices for CIFAR‐10 classification. Left: Digital‐only double‐layer convolutional network, achieving 86.6% test accuracy. Right: Full hybrid pipeline (optical front end + lightweight digital classifier), attaining 85.7% accuracy. (d) Representative input cytological images from the SIPaKMeD dataset, a high‐resolution medical database of cervical cell morphology for Pap smear screening. Images contain fine nuclear and cytoplasmic details critical for diagnostic classification. Scale bar: 128 pixels. (e) Optically convolved feature maps corresponding to the images in (d). The fixed Bessel‐vortex first layer extracts diagnostically relevant edge and curvature features, while the tunable second‐layer vortex matrix adaptively weights these priors. Note the preservation of subtle chromatin texture in the *l* = 2 and *l* = 3 channels. (f) Confusion matrices for SIPaKMeD five‐class cell classification. Left: Digital‐only double‐layer convolutional network, achieving 99.6% test accuracy. Right: Full hybrid pipeline with online error correction, reaching 99.3% accuracy. (g) Online training convergence curves for the SIPaKMeD classification task. Simulated (purple) and experimentally measured (blue) cross‐entropy loss trajectories over 15 training epochs. (h) Learned second‐layer convolution kernel weights for the three‐color channels (R, G, B) across the four polarization‐multiplexed Bessel‐vortex modes (*l* = 0,1,2,3). (i) t‐distributed stochastic neighbor embedding (t‐SNE) visualization of feature representations after the optical convolutional front end for the SIPaKMeD dataset.

To rigorously assess the meta‐imager's capacity for high‐precision, domain‐specific adaptation, we turned to the SIPaKMeD dataset—a publicly available collection of high‐resolution (128 × 128 pixel) cervical cytology images annotated for five diagnostic cell categories (superficial–intermediate, parabasal, koilocytotic, dyskeratotic, and metaplastic). Accurate classification of these cellular morphologies is critical for automated Pap smear screening, yet the subtle textural and architectural cues that distinguish categories pose a formidable challenge to shallow classifiers. Representative input images and their corresponding optically convolved feature maps are displayed in Figure 5d,e, respectively. Qualitatively, the meta‐imager's Bessel‐vortex first layer amplifies diagnostically salient structures—nuclear membrane contours, chromatin granularity, and cytoplasmic boundaries—while the tunable second‐layer vortex matrix adaptively gates these features according to the learned kernel weights. Notably, the *l* = 2 and *l* = 3 channels, which implement higher‐order spatial differentiation, reveal fine textural modulations that are barely perceptible in the raw pixel data.

The classification results for SIPaKMeD are summarized in the confusion matrices of Figure 5f. A digital single‐layer convolutional network achieves a baseline accuracy of 83.6%, reflecting the inherent difficulty of the task given the constrained model capacity. Remarkably, the hybrid meta‐imager pipeline—after undergoing the closed‐loop online training procedure detailed in Figure 3—reaches a final test accuracy of 99. 3%. This 15.7 percentage‐point absolute improvement is among the highest reported for optical front‐end acceleration on a medical imaging benchmark and underscores the transformative impact of embedding two effective convolutional layers within a single physical aperture.

The convergence dynamics of online training are plotted in Figure 5g. Both simulated and experimentally measured loss trajectories exhibit rapid descent within the first five epochs, plateauing at nearly identical final cross‐entropy values. The close correspondence between simulation and experiment validates the accuracy of our optical forward model and confirms that the amplitude‐driven gradient updates, backpropagated through the APNN and applied to the LC transmission map, effectively steer the physical system toward the loss basin of the ideal digital model. Figure 5h displays the normalized 3 × 3 convolution kernel weight for all combinations of color channel (R, G, B) and polarization mode (*l* = 0,1,2,3). Minor channel‐dependent differences reflect residual chromatic dispersion, which is automatically compensated during online training. Finally, Figure 5i provides an intuitive visualization of the feature‐space transformation effected by the meta‐imager. t‐distributed stochastic neighbor embedding (t‐SNE) projections of the raw SIPaKMeD pixel data reveal substantial inter‐class overlap, with the five diagnostic categories largely intermixed in the high‐dimensional input space (Note S16 and Figure S17). Following optical convolution, the same cell images project to well‐separated clusters with markedly improved intra‐class compactness. Collectively, these results establish the spatiotemporal intrinsic depth meta‐imager as a versatile, high‐performance front end for energy‐efficient machine vision across domains spanning natural imagery to high‐resolution medical diagnostics.

## 3 Conclusions

We have proposed and experimentally demonstrated a single‐layer metasurface imager endowed with spatiotemporal intrinsic depth—a design paradigm that collapses two complete convolutional layers into a single sub‐millimeter‐thick flat optic. By leveraging polarization‐division multiplexing, the metasurface encodes four fixed Bessel‐vortex channels (*l* = 0, 1, 2, 3) as an immutable first convolutional layer, with the *l* = 0 channel physically implementing a hardware‐native residual connection. A reconfigurable second layer is realized through a metasurface‐generated 3 × 3 optical vortex matrix, whose weights are dynamically tuned via time‐sequential liquid‐crystal amplitude modulation. This temporal differential encoding scheme resolves the long‐standing sign constraint of incoherent optical computing while uniquely enabling in situ training that compensates for nanofabrication imperfections. The broadband dispersion engineering of the Bessel‐vortex basis further sustains convolution fidelity across the broadband spectrum, extending the operational scope to full‐color imagery. Our experimental validation across three complementary benchmarks—rotation‐robust fashion‐item classification, full‐color CIFAR‐10 recognition, and high‐resolution SIPaKMeD medical cytology—demonstrates that the meta‐imager boosts single‐layer digital baseline accuracies by substantial margins (from 57.5% to 86.6% on CIFAR‐10, and from 83.6% to 99.3% on SIPaKMeD) while offloading approximately 99.7% of the multiply–accumulate operations to the optical domain. The closed‐loop online training procedure, driven by an amplitude feedback neural network, converges within a few epochs and yields learned kernels that automatically absorb static phase errors introduced during fabrication.

This work establishes a new paradigm for edge‐intelligent machine vision by demonstrating that the expressive capacity of multi‐layer diffractive networks can be folded into a monolithic metasurface through the orthogonal multiplexing dimensions of polarization and time. The decoupled amplitude–phase architecture—wherein a liquid‐crystal layer provides reconfigurable weight control and an all‐dielectric metasurface delivers high‐precision phase engineering—overcomes the efficiency–accuracy trade‐off that has constrained prior metasurface processors. More broadly, the concept of spatiotemporal intrinsic depth suggests a pathway toward ultra‐compact, task‐specific optical front ends that shift the dominant fraction of neural network inference to the passive, high‐speed physical layer. Future extensions may include scaling to larger kernel arrays, integration with advanced photodetector technologies for on‐chip polarization sorting, and co‐design with quantized digital back ends for fully integrated intelligent sensors in autonomous systems, wearable diagnostics, and augmented reality.

### 3.1 Metasurface Fabrication

The metasurface was fabricated on a 500‐µm‐thick fused silica (JGS2) substrate. A 750‐nm‐thick hydrogenated amorphous silicon film was deposited by plasma‐enhanced chemical vapor deposition (PECVD) at 300°C using silane and argon, achieving a deposition rate of approximately 40 nm min^−1^. The metasurface pattern was defined in a ZEP520A electron‐beam resist by electron‐beam lithography operating at 100 kV with an exposure dose of 270 µC/cm^2^. After development, a 100‐nm‐thick chromium hard mask was deposited via magnetron sputtering and subsequently patterned by lift‐off in acetone and isopropanol. The pattern was transferred into the amorphous silicon layer by inductively coupled plasma reactive ion etching with a gas mixture. Finally, the residual chromium mask was removed using a commercial chromium etchant, yielding an all‐dielectric metasurface with subwavelength periodicity. Fabrication fidelity was verified by scanning electron microscopy at multiple stages.

## 4 Method

We evaluate our model on three public benchmark datasets: Fashion‐MNIST, CIFAR‐10, and SIPaKMeD. Samples are programmatically loaded via the `torchvision` Python library. Representative visualized test samples in figures are official samples from each public dataset release.

Original dataset source links:

CIFAR‐10: https://www.cs.toronto.edu/~kriz/cifar.html

Fashion‐MNIST: https://github.com/zalandoresearch/fashion‐mnist

SIPaKMeD: https://cs.uoi.gr/~marina/sipakmed.html

No new raw data were generated in this manuscript.

### 4.1 Digital Neural Network Training

The experimental evaluation was performed on three complementary benchmarks: Fashion‐MNIST for rotation‐robustness validation, CIFAR‐10 for broadband color imaging assessment, and SIPaKMeD for online training and error‐correction verification. The meta‐imager operates with four fixed polarization‐multiplexed Bessel‐vortex channels (topological charges *l* = 0, 1, 2, 3), each of which is further modulated by a reconfigurable optical vortex matrix via temporally sequenced liquid‐crystal amplitude control. Positive and negative kernel weights are physically realized through differential frame acquisition, wherein two successive intensity distributions corresponding to complementary transmission maps are digitally subtracted. The hybrid optoelectronic pipeline was implemented in PyTorch with CUDA acceleration, and all digital back‐end training employed the Adam optimizer to minimize categorical cross‐entropy loss via automatic differentiation. The amplitude feedback neural network, a lightweight encoder–decoder trained offline on 4000 PSF–transmittance pairs, generated initial LC transmission maps within sub‐millisecond latency, after which online fine‐tuning proceeded for a limited number of epochs to compensate for residual fabrication errors and task‐specific feature shifts.

## Author Contributions

**Mengguang Wang**: conceptualization; methodology; software; dataCuration; investigation; validation; formalAnalysis; supervision; projectAdministration; resources; writingReviewEditing; writingOriginalDraft. **Chenglong Zheng**: software. **Huaping Zang**: dataCuration. **Changwei Zhang**: investigation. **Huai Xia**: validation. **Chang Wang**: fundingAcquisition. **Zhenrong Zheng**: fundingAcquisition; writing Review Editing; resources; visualization.

## Funding

This work was supported by National Natural Science Foundation of China (62305092), National Key Research and Development Program of China (2022YFF0705500), National Natural Science Foundation of China (62401511) and Key R&D and Promotion Special (Scientific Problem Tackling) Project of Henan Province (262102211120).

## Conflicts of Interest

The authors declare no conflicts of interest.

## Supporting information




**Supporting File**: advs78340‐sup‐0001‐SuppMat.pdf.

## Data Availability

The data that support the findings of this study are available from the corresponding author upon reasonable request.

## References

[1] Y. LeCun , Y. Bengio , and G. Hinton , “Deep Learning,” Nature 521 (2015): 436–444. 10.1038/nature14539 26017442

[2] A. Krizhevsky , I. Sutskever , and G. E. Hinton , “ImageNet Classification with Deep Convolutional Neural Networks,” Communications of the ACM 60 (2017): 84–90.

[3] L. Liu , S. Lu , R. Zhong , et al., “Computing Systems for Autonomous Driving: State of the Art and Challenges,” IEEE Internet of Things Journal 8 (2021): 6469–6486.

[4] L. Mennel , J. Symonowicz , S. Wachter , D. K. Polyushkin , A. J. Molina‐Mendoza , and T. Mueller , “Ultrafast Machine Vision with 2D Material Neural Network Image Sensors,” Nature 579 (2020): 62–66. 10.1038/s41586-020-2038-x 32132692

[5] W. Shi , Z. Huang , H. Huang , et al., “LOEN: Lensless Opto‐Electronic Neural Network Empowered Machine Vision,” Light: Science & Applications 11 (2022): 121. 10.1038/s41377-022-00809-5 PMC9068799 35508469

[6] G. Wetzstein , A. Ozcan , S. Gigan , et al., “Inference in Artificial Intelligence with Deep Optics and Photonics,” Nature 588 (2020): 39–47. 10.1038/s41586-020-2973-6 33268862

[7] T. Wang , S.‐Y. Ma , L. G. Wright , T. Onodera , B. C. Richard , and P. L. McMahon , “An Optical Neural Network Using Less than 1 Photon per Multiplication,” Nature Communications 13 (2022): 123. 10.1038/s41467-021-27774-8 PMC8748769 35013286

[8] T. Wang , M. M. Sohoni , L. G. Wright , et al., “Image Sensing with Multilayer Nonlinear Optical Neural Networks,” Nature Photonics 17 (2023): 408–415.

[9] M. Qiu , W. Zheng , J. Chen , Z. Cheng , L. Wang , and Q. Wang , “Highly Precise Optical Positioning through Deep Learning‐Assisted Photo‐Thermoelectric Detection,” Applied Physics Letters 124 (2024): 261104.

[10] Y. Peng and W. Chen , “Deep Learning‐Enhanced Ghost Imaging through Dynamic and Complex Scattering Media with Supervised Corrections of Dynamic Scaling Factors,” Applied Physics Letters 124 (2024): 181104.

[11] T. Zhou , X. Lin , J. Wu , et al., “Large‐Scale Neuromorphic Optoelectronic Computing with a Reconfigurable Diffractive Processing Unit,” Nature Photonics 15 (2021): 367–373.

[12] J. Chang , V. Sitzmann , X. Dun , W. Heidrich , and G. Wetzstein , “Hybrid Optical‐Electronic Convolutional Neural Networks with Optimized Diffractive Optics for Image Classification,” Scientific Reports 8 (2018): 12324. 10.1038/s41598-018-30619-y PMC6098044 30120316

[13] X. Lin , Y. Rivenson , N. T. Yardimci , et al., “All‐Optical Machine Learning Using Diffractive Deep Neural Networks,” Science 361 (2018): 1004–1008. 10.1126/science.aat8084 30049787

[14] Y.‐H. Chen , T. Krishna , J. S. Emer , and V. Sze , “Eyeriss: An Energy‐Efficient Reconfigurable Accelerator for Deep Convolutional Neural Networks,” IEEE Journal of Solid‐State Circuits 52 (2017): 127–138.

[15] X. Xu , M. Tan , B. Corcoran , et al., “11 TOPS Photonic Convolutional Accelerator for Optical Neural Networks,” Nature 589 (2021): 44–51. 10.1038/s41586-020-03063-0 33408378

[16] J. Feldmann , N. Youngblood , M. Karpov , et al., “Parallel Convolutional Processing Using an Integrated Photonic Tensor Core,” Nature 589 (2021): 52–58. 10.1038/s41586-020-03070-1 33408373

[17] X. Luo , Y. Hu , X. Ou , et al., “Metasurface‐Enabled on‐Chip Multiplexed Diffractive Neural Networks in the Visible,” Light: Science & Applications 11 (2022): 158. 10.1038/s41377-022-00844-2 PMC9142536 35624107

[18] Z. Xue , T. Zhou , Z. Xu , S. Yu , Q. Dai , and L. Fang , “Fully Forward Mode Training for Optical Neural Networks,” Nature 632 (2024): 280–286. 10.1038/s41586-024-07687-4 PMC11306102 39112621

[19] Z. Xu , T. Zhou , M. Ma , C. Deng , Q. Dai , and L. Fang , “Large‐Scale Photonic Chiplet Taichi Empowers 160‐TOPS/W Artificial General Intelligence,” Science 384 (2024): 202–209. 10.1126/science.adl1203 38603505

[20] K. Cui , S. Rao , S. Xu , et al., “Spectral Convolutional Neural Network Chip for In‐Sensor Edge Computing of Incoherent Natural Light,” Nature Communications 16 (2025): 81. 10.1038/s41467-024-55558-3 PMC11696300 39747892

[21] B. Chen , X. Sun , X. Li , et al., “4H‐SiC Metalens: Mitigating Thermal Drift Effect in High‐Power Laser Irradiation,” Advanced Materials 37 (2025): 2412414. 10.1002/adma.202412414 39558784

[22] M. Wang , Y. Li , H. Li , et al., “Ultrawide‐FOV Full‐Color Waveguides Free of Rainbow and Fishbone Artifacts via Physics‐Constrained Generative Metagrating Design,” Science Advances 12 (2026): aeh5234. 10.1126/sciadv.aeh5234 PMC13488895 42616859

[23] Q. Fan , M. Liu , C. Zhang , et al., “Independent Amplitude Control of Arbitrary Orthogonal States of Polarization via Dielectric Metasurfaces,” Physical Review Letters 125 (2020): 267402. 10.1103/PhysRevLett.125.267402 PMC8143859 33449781

[24] Q. Fan , W. Xu , X. Hu , et al., “Trilobite‐Inspired Neural Nanophotonic Light‐Field Camera with Extreme Depth‐of‐Field,” Nature Communications 13 (2022): 2130. 10.1038/s41467-022-29568-y PMC9019092 35440101

[25] X. Liu , J. Zhang , B. Leng , et al., “Edge Enhanced Depth Perception with Binocular Meta‐Lens,” Opto‐Electronic Science 3 (2024): 230033.

[26] J. Cheng , Y. Zhou , Y. Gao , et al., “Quantitative Phase Imaging for Meta‐Lenses by Phase Retrieval,” Advanced Optical Materials 13 (2025): 2402833.

[27] J. Cheng , Z. Geng , Y. Zhou , et al., “Tunable Meta‐Device for Large Depth of Field Quantitative Phase Imaging,” Nanophotonics 14 (2025): 1249–1256. 10.1515/nanoph-2024-0661 PMC12019949 40290285

[28] B. Xiong , Y. Liu , Y. Xu , et al., “Breaking the Limitation of Polarization Multiplexing in Optical Metasurfaces with Engineered Noise,” Science 379 (2023): 294–299. 10.1126/science.ade5140 36656947

[29] H. Kwon , E. Arbabi , S. M. Kamali , M. Faraji‐Dana , and A. Faraon , “Single‐Shot Quantitative Phase Gradient Microscopy Using a System of Multifunctional Metasurfaces,” Nature Photonics 14 (2020): 109–114.

[30] N. Yu , P. Genevet , M. A. Kats , et al., “Light Propagation with Phase Discontinuities: Generalized Laws of Reflection and Refraction,” Science 334 (2011): 333–337. 10.1126/science.1210713 21885733

[31] R. Hu , Q. Min , X. Liu , A. Dai , J. Guo , and G. Situ , “Terahertz Programmable Metasurface for Phase Modulation Based on Free Carrier Plasma Dispersion Effect,” Applied Physics Letters 124 (2024): 251703.

[32] K. He , Q. Niu , Y. Xie , S. Xiao , Y. Yang , and J. Zhu , “Bound State in the Continuum (BIC) Excited by the Metasurface Breaking in‐Plane Symmetry and Quasi‐BIC for Terahertz Sensing,” Applied Physics Letters 124 (2024): 231702.

[33] Q. Zhang , Z. Yu , X. Liu , C. Wang , and Z. Zheng , “End‐to‐End Joint Optimization of Metasurface and Image Processing for Compact Snapshot Hyperspectral Imaging,” Optics Communications 530 (2023): 129154.

[34] Q. Zhang , P. Lin , C. Wang , et al., “Neural‐Optic Co‐Designed Polarization‐Multiplexed Metalens for Compact Computational Spectral Imaging,” Laser & Photonics Reviews 18 (2024): 2400187.

[35] M. Wang , Q. Zhang , X. Liu , et al., “Multichannel Meta‐Imagers Based on Electrically Tunable Metasurfaces for Accelerating Matrix Operations,” Optics Express 32 (2024): 39915–39923. 10.1364/OE.537622 39573799

[36] H. Zheng , Q. Liu , I. I. Kravchenko , X. Zhang , Y. Huo , and J. G. Valentine , “Multichannel Meta‐Imagers for Accelerating Machine Vision,” Nature Nanotechnology 19 (2024): 471–478. 10.1038/s41565-023-01557-2 PMC11031328 38177276

[37] C. Guo , M. Xiao , M. Minkov , Y. Shi , and S. Fan , “Photonic Crystal Slab Laplace Operator for Image Differentiation,” Optica 5 (2018): 251–256.

[38] M. Wang , J. Wang , C. Zhang , et al., “Self‐Tunable Metasurface Photoelectric Hybrid Neural Network,” Laser & Photonics Reviews 20 (2026): 02006.

[39] H. Wang , C. Guo , Z. Zhao , and S. Fan , “Compact Incoherent Image Differentiation with Nanophotonic Structures,” ACS Photonics 7 (2020): 338–343.

[40] X. Zhang , B. Bai , H.‐B. Sun , G. Jin , and J. Valentine , “Incoherent Optoelectronic Differentiation Based on Optimized Multilayer Films,” Laser & Photonics Reviews 16 (2022): 2200038.

[41] H. Zheng , Q. Liu , Y. Zhou , I. I. Kravchenko , Y. Huo , and J. Valentine , “Meta‐Optic Accelerators for Object Classifiers,” Science Advances 8 (2022): abo6410. 10.1126/sciadv.abo6410 PMC9328681 35895828

[42] C. Liu , Q. Ma , Z. J. Luo , et al., “A Programmable Diffractive Deep Neural Network Based on a Digital‐Coding Metasurface Array,” Nature Electronics 5 (2022): 113–122.

[43] Y. Hu , X. Ou , T. Zeng , et al., “Electrically Tunable Multifunctional Polarization‐Dependent Metasurfaces Integrated with Liquid Crystals in the Visible Region,” Nano Letters 21 (2021): 4554–4562. 10.1021/acs.nanolett.1c00104 34047184

[44] X. Ou , T. Zeng , Y. Zhang , et al., “Tunable Polarization‐Multiplexed Achromatic Dielectric Metalens,” Nano Letters 22 (2022): 10049–10056. 10.1021/acs.nanolett.2c03798 36473130

[45] L. Huang , Q. A. A. Tanguy , J. E. Fröch , S. Mukherjee , K. F. Böhringer , and A. Majumdar , “Photonic Advantage of Optical Encoders,” Nanophotonics 13 (2024): 1191–1196. 10.1515/nanoph-2023-0579 PMC11501926 39634009

[46] Z. Fang , R. Chen , J. E. Fröch , et al., “Nonvolatile Phase‐Only Transmissive Spatial Light Modulator with Electrical Addressability of Individual Pixels,” ACS Nano 18 (2024): 11245–11256. 10.1021/acsnano.4c00340 38639708

[47] G. K. Shirmanesh , R. Sokhoyan , P. C. Wu , and H. A. Atwater , “Electro‐Optically Tunable Multifunctional Metasurfaces,” ACS Nano 14 (2020): 6912–6920. 10.1021/acsnano.0c01269 32352740

