# Supplementary material for: Spatiotemporal Computational Meta‐Imager for Broadband Dual‐Layer Optical Convolution
Source: Adv Sci (Weinh). 2026 Oct 18:e78340. Online ahead of print. doi: 10.1002/advs.78340 (PMC13677043; doi:10.1002/advs.78340)
Supplement: Supplementary file 1 [file ADVS-9999-e78340-s001.pdf]

Spatiotemporal computational meta-imager for broadband dual-layer optical convolution

Mengguang Wang[1], Chenglong Zheng[2], Huaping Zang[2], Changwei Zhang[1], Huai Xia[1], Chang Wang[3], and Zhenrong Zheng[1,*]

[1]College of Optical Science and Engineering, Zhejiang University, Hangzhou 310027, China

[2]Key Laboratory of Material Physics, Ministry of Education, School of Physics, Zhengzhou University, Zhengzhou 450001, China

[3]Zhangjiang Laboratory, Shanghai 201210, China

Correspondence: zzr@zju.edu.cn (Z. Zheng)

Contents

S1. Three experimental data sets 3

S2. The design of the Bessel vortex 4

S3. The design of the vortex array 5

S4. Floating-point Operations in Convolutional Neural Network 7

S5. Polarization multiplexing 9

S6. Phase Response of Rectangle Nanopillars 13

S7. Amplitude prediction neural network (APNN) 15

S8. Optical Characterization Setup for Point Spread Function 18

S9. Experiment on wide-band PSF 19

S10. The weight distribution of the three experiments 20

S11. Optical Characterization Setup for Object Classification 22

S12. Feature mapping of Fashion-MNIST dataset 24

S13. Feature mapping of CIFAR-10 dataset 25

S14. Feature mapping of SIPaKMeD dataset 26

S15. Confusion matrices for three classification architectures across benchmark datasets 27

S16. Prediction accuracy analysis for Fashion MNIST dataset, CIFAR-10, and SIPaKMeD. 31

References 32

S1. Three experimental data sets

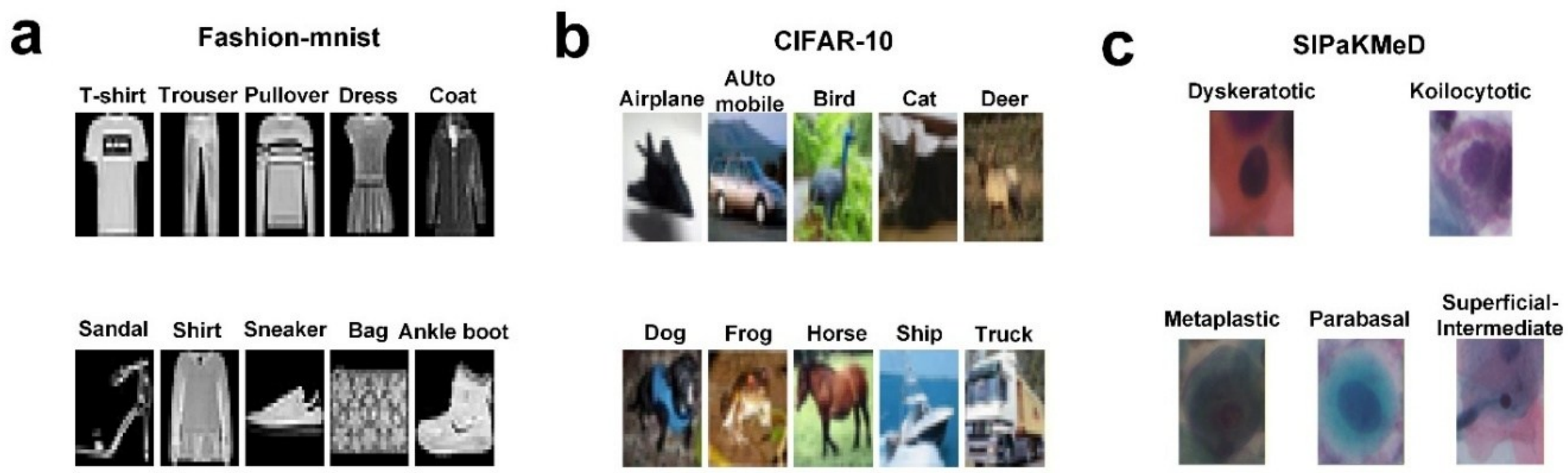

Figure S1. Representative samples from the three benchmark datasets used for experimental validation. (a) Fashion-MNIST dataset. Grayscale images of 10 clothing categories, each with a spatial resolution of 28 × 28 pixels. (b) CIFAR-10 dataset. Full-color variant of CIFAR-10 comprising 10 object categories (airplane, automobile, bird, cat, deer, dog, frog, horse, ship, truck) with 32 × 32 pixel resolution and three RGB color channels. (c) SIPaKMeD dataset. High-resolution cervical cytology images (128 × 128 pixels) annotated for five diagnostic cell types: superficial–intermediate, parabasal, koilocytotic, dyskeratotic, and metaplastic. The dataset was employed for online training and fabrication-error compensation validation. Representative test samples are sourced from the public Fashion-MNIST, CIFAR-10, and SIPaKMeD benchmark datasets.

The experimental validation of the spatiotemporal intrinsic depth meta-imager was conducted on three publicly available datasets spanning diverse imaging modalities and application domains. Representative samples from each dataset are displayed in Supplementary Fig. S1.

S2. The design of the Bessel vortex

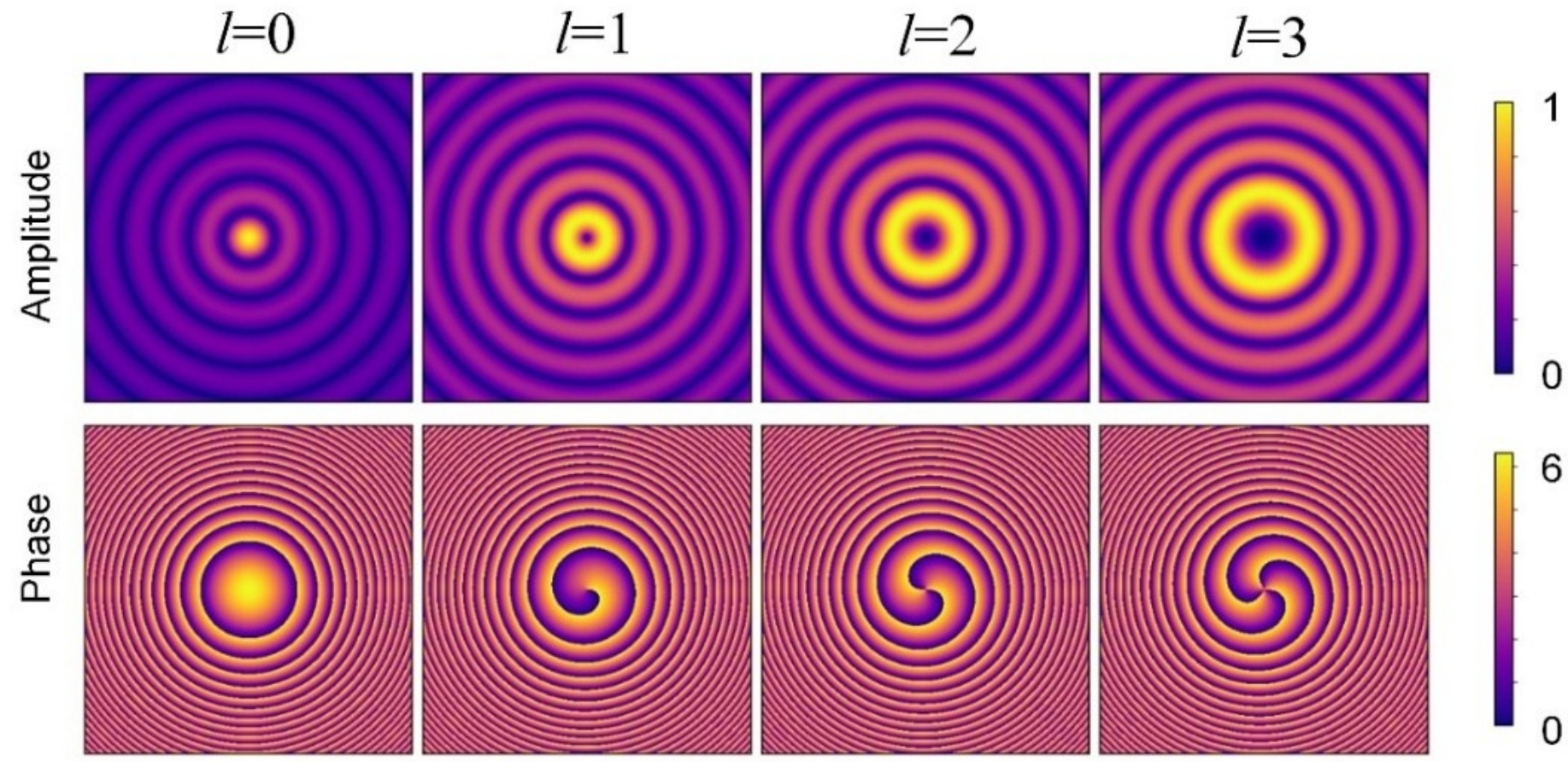

Figure S2. Amplitude and phase profiles of the polarization-multiplexed Bessel-vortex basis.

Top row: Normalized amplitude distributions of the four Bessel-vortex channels with topological charges l=0,1,2,3 at the metasurface plane. The radial envelope follows the l-th order Bessel function of the first kind, $J_l(\alpha r)$, exhibiting a central maximum for l=0 and characteristic ring-like profiles with central nulls for higher orders. Bottom row: Corresponding phase profiles over the same spatial aperture. The phase comprises the azimuthal vortex term $e^{il\phi}$ and a quadratic focusing term $e^{-ik_0\frac{r^2}{2f}}$, producing the spiral phase ramps and concentric Fresnel rings visible in the color-coded phase maps. These four amplitudes–phase pairs are encoded into orthogonal polarization channels via metasurface design, forming the immutable first convolutional layer of the meta-imager[1].

S3. The design of the vortex array

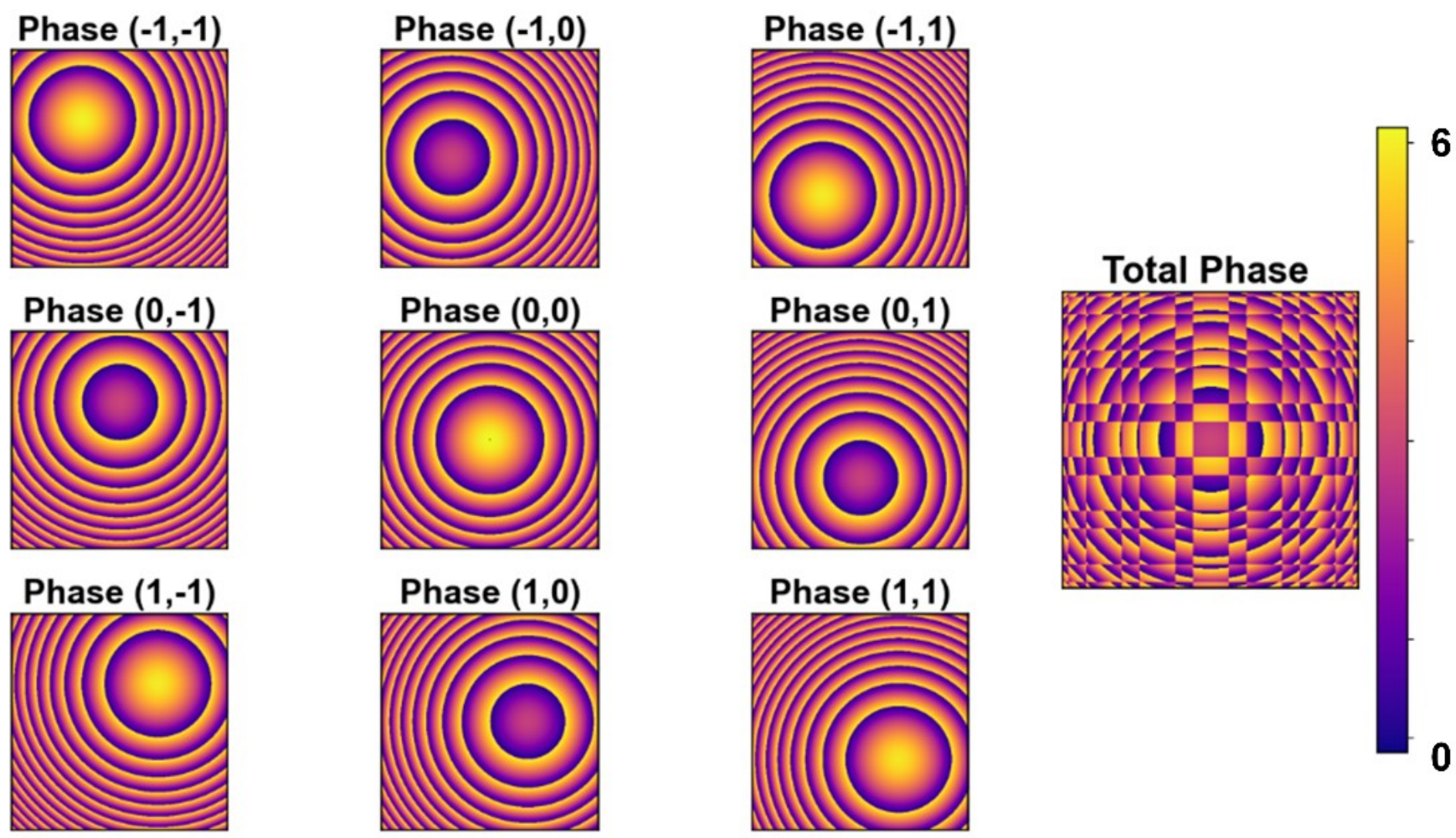

Figure S3. The design of the vortex array.

The second convolutional layer of the meta-imager is physically realized by engineering the point spread function (PSF) of the metasurface into a discrete 3×3 array of diffraction-limited focal spots, each carrying an independent vortex phase[2].

While the generation of a discrete focal-spot array using a metasurface has been demonstrated previously (Ref. 36), our implementation introduces two key innovations beyond that prior work. First, the 3×3 vortex array here is cascaded with a polarization-multiplexed Bessel–vortex first layer, forming a factorized dual-layer kernel that is not a single-shot convolution but a structured decomposition with enhanced parametric expressivity. Second, the intensity weights of the vortex array are dynamically reconfigurable via an LC amplitude modulator and are updated via closed-loop online training, enabling task-specific adaptation and fabrication-error compensation—a

capability not present in the static metasurface of Ref. 36.

S4. Floating-point Operations in Convolutional Neural Network

Operations		FLOPs
MODEL	Convolution1	2×3×Channel×Kernel shape ×Output shape =194400
	Convolution2	2×4×Channel×Kernel shape ×Output shape =508032
	Max Pooling	Channel×Data Height×Data Width =1764
	ReLU	Input Neurons = 7056
	Fully Connected Layer	2×Input size×Output size= 180

Table S1. Floating-point Operations of Each Layer in the Neural Network.

In a conventional digital convolutional neural network, the convolution layers account for the dominant fraction of the total floating-point operations, with the computational cost scaling quadratically with kernel size and linearly with spatial resolution. In the proposed optoelectronic hybrid neural network, the fixed first-layer Bessel-vortex priors and the reconfigurable second-layer optical vortex array collectively replace the most arithmetic-intensive convolution stages. A detailed operation count reveals that the optoelectronic front end executes approximately 99.7% of the multiply–accumulate operations that would otherwise be performed digitally for an equivalent single-layer convolutional architecture. This substantial offloading is achieved passively through free-space optical propagation, requiring no additional power for the convolution arithmetic itself. Consequently, the digital back end is reduced to lightweight post-processing—comprising only pooling, activation, and a fully connected classification layer—

thereby enabling ultra-rapid inference and significantly reduced energy consumption per recognition task[3,4].

S5. Polarization multiplexing

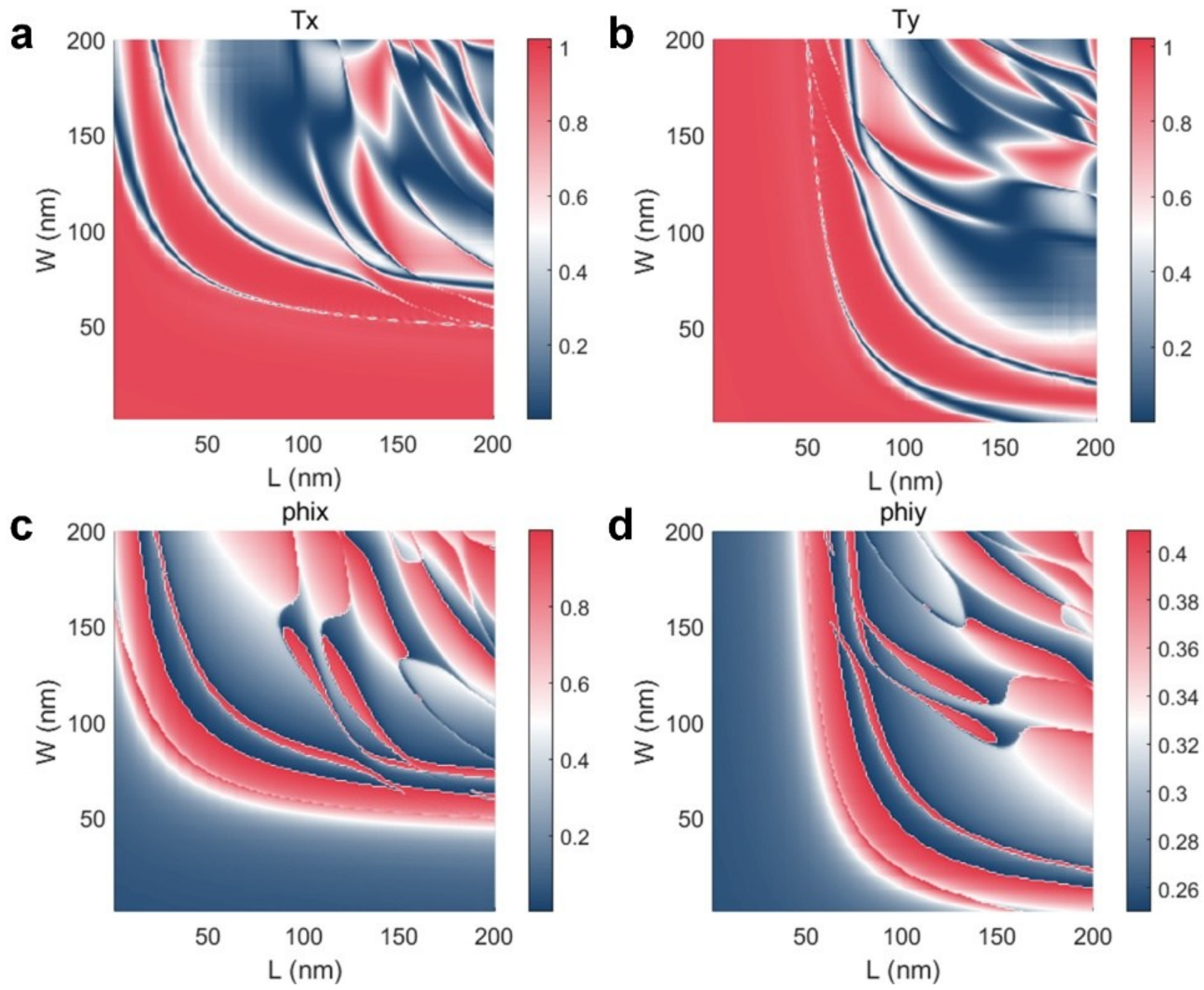

Figure S4. Birefringence of a rectangular nanopillar. L and W denote the length and width of the rectangular nanopillar, respectively. The nanopillar is fabricated from silicon and exhibits effective birefringence. (a) and (b) show the transmittance variations when x-polarized and y-polarized light waves propagate through the rectangular nanopillar. Tx and Ty indicate the amplitude transmission coefficients for x-polarized and y-polarized light, respectively. (c) and (d) display the corresponding phase changes. ϕ_x and ϕ_y represent the normalized phase of x-polarized and y-polarized light, respectively. The simulations assume an operating wavelength of 950 nm, a lattice constant of 520 nm, and a silicon nanopillar height of 750 nm.

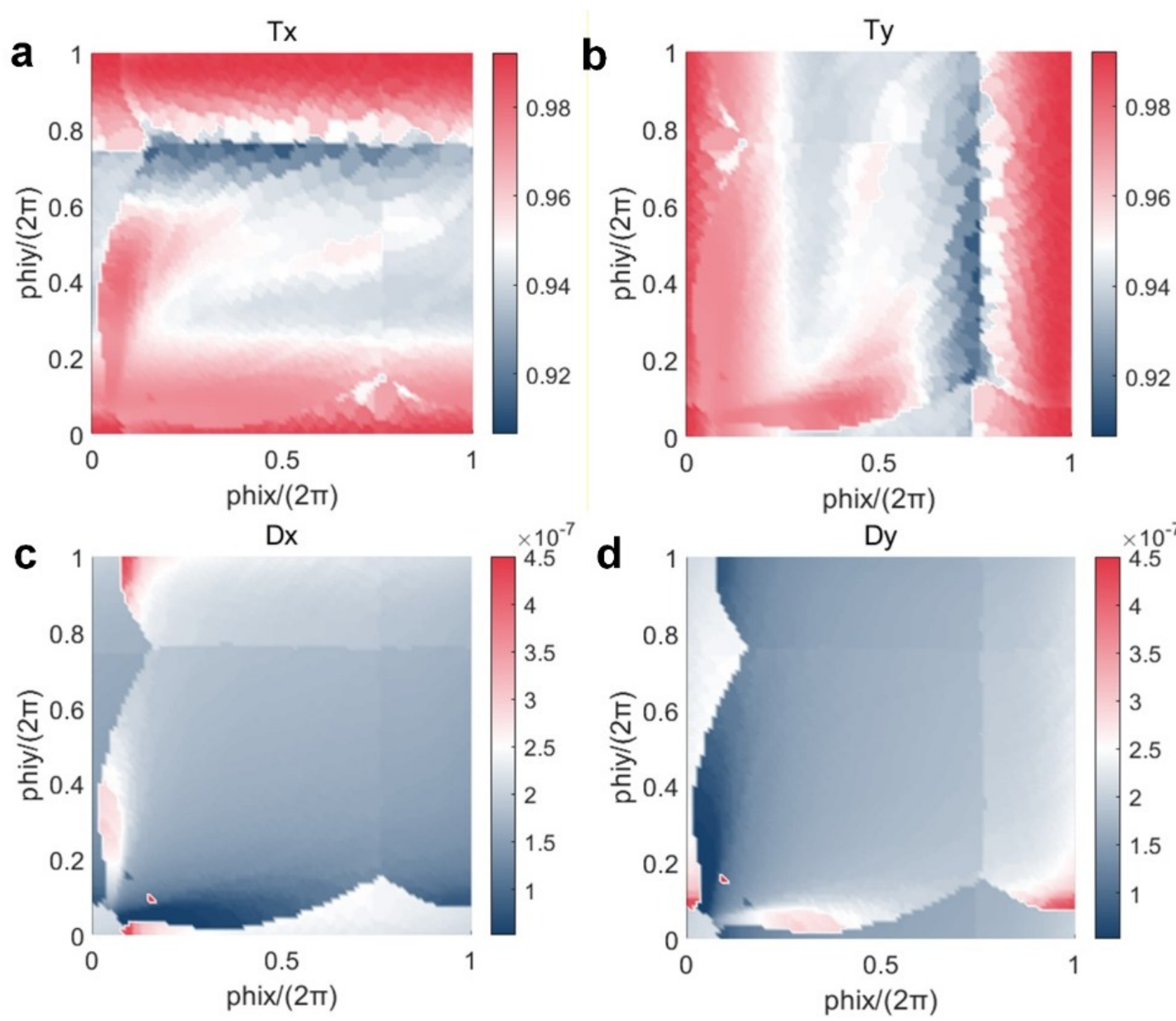

Figure S5. (a)-(b) Simulated colour-coded values of the intensity transmission coefficients corresponding to the choice of rectangular diameters. (c)-(d) Simulated colour-coded values of the rectangular post diameters (Dx and Dy) for achieving phix and phiy phase shifts for x- and y-polarized optical waves, respectively.

To realize polarization multiplexing, a comprehensive meta-atom database was constructed by scanning rectangular silicon nanocolumns. Each atom in this database acts as a half-wave plate with a phase response constraint of $\phi_x - \phi_y = \pi$[5,6]. Here, ϕ_x and ϕ_y represent the phase responses under x-polarized and y-polarized light excitation, respectively, with a specific rotation angle, θ. The polarization-dependent behavior of these meta-atoms can be rigorously described by the Jones Matrix, which is given by:

$$\begin{bmatrix} E_x \\ E_y \end{bmatrix} = \begin{bmatrix} \cos(\theta) & \sin(\theta) \\ -\sin(\theta) & \cos(\theta) \end{bmatrix} \begin{bmatrix} e^{i\phi_x} & 0 \\ 0 & e^{i\phi_x+\pi} \end{bmatrix} \begin{bmatrix} \cos(\theta) & -\sin(\theta) \\ \sin(\theta) & \cos(\theta) \end{bmatrix} \begin{bmatrix} 1 \\ 0 \end{bmatrix} \tag{S1}$$

The matrix $[1,0]^T$ represents linearly polarized illumination that simultaneously incorporates both left circularly polarized (LCP) and right circularly polarized (RCP) components with equal intensity. Equation.S1(Eq. S1) can thus be simplified as follows:

$$\begin{bmatrix} E_x \\ E_y \end{bmatrix} = e^{i\phi_x} \begin{bmatrix} \cos^2(\theta) - \sin^2(\theta) \\ -2\cos(\theta)\sin(\theta) \end{bmatrix} \tag{S2}$$

To separate the circular polarization components, quarter-wave plates are characterized using the Jones Matrix, as follows:

$$M_{QWP} = \begin{bmatrix} \cos(\pi/4) & \sin(\pi/4) \\ -\sin(\pi/4) & \cos(\pi/4) \end{bmatrix} \begin{bmatrix} e^{i\pi/2} & 0 \\ 0 & 1 \end{bmatrix} \begin{bmatrix} \cos(\pi/4) & -\sin(\pi/4) \\ \sin(\pi/4) & \cos(\pi/4) \end{bmatrix} \tag{S3}$$

The LCP and RCP electric field can be described as follows:

$$\begin{aligned} \begin{bmatrix} E_{LCP} \\ E_{RCP} \end{bmatrix} &= M_{QWP} \begin{bmatrix} E_x \\ E_y \end{bmatrix} \\ &= \frac{1}{2} e^{i\phi_x} \left\{ \begin{bmatrix} \cos(2\theta) - \sin(2\theta) \\ \cos(2\theta) - \sin(2\theta) \end{bmatrix} + i \begin{bmatrix} \cos(2\theta) + \sin(2\theta) \\ -\cos(2\theta) - \sin(2\theta) \end{bmatrix} \right\} \end{aligned} \tag{S4}$$

By further simplifying Eq. S4, we can get the following:

$$\begin{bmatrix} E_{LCP} \\ E_{RCP} \end{bmatrix} = \frac{\sqrt{2}}{2} e^{i\phi_x} \begin{bmatrix} e^{i\phi_\theta} \\ e^{-i\phi_\theta} \end{bmatrix} \tag{S5}$$

where ϕ_θ can be defined by the following equation:

$$\phi_\theta = \operatorname{atan}\left[\frac{\cos(2\theta) + \sin(2\theta)}{\cos(2\theta) - \sin(2\theta)}\right] = \left[2\theta + \frac{\pi}{4}\right] \tag{S6}$$

Combining Eq. S5 and Eq. S6, the resulting output amplitude remains unity. The phase response, however, varies according to the different states of circular polarization and can be expressed as follows:

$$\begin{bmatrix}\phi_{LCP}\\ \phi_{RCP}\end{bmatrix} = \begin{bmatrix}\phi_x + 2\theta + \pi/4\\ \phi_x - 2\theta - \pi/4\end{bmatrix} \tag{S7}$$

Therefore, the phase delay for orthogonal circularly polarized light can be independently manipulated by adjusting the width, length, and rotation angle of the meta-atoms[7].

Our metasurface design jointly employs propagation phase (via the length L and width W of each rectangular nanopillar) and geometric (Pancharatnam–Berry) phase (via the in-plane rotation angle θ). By satisfying the half-waveplate condition $\varphi_x - \varphi_\gamma = \pi$, the phase responses for left- and right-handed circular polarizations are independently controlled as φ_LCP = $\varphi_x + 2\theta + \pi/4$ and φ_RCP = $\varphi_x - 2\theta - \pi/4$. The meta-atom parameters are: amorphous silicon nanopillars on fused silica, period 520 nm, height 750 nm, design wavelength 950 nm, with L and W selected from a pre-computed library to provide high transmission (>70%) and full 2π phase coverage.

We carefully considered two implementation approaches. The first uses a single metasurface that deflects different polarizations to distinct spatial angles, achieving four-channel separation in one aperture. However, this inevitably introduces residual polarization crosstalk due to imperfect phase control and fabrication errors, compromising the independence of the four filtering operations. To avoid this limitation, we adopted a second approach in our experimental prototype: two separate metalens regions—one optimized for linear polarization multiplexing and the other for circular polarization multiplexing. The measured polarization extinction ratio exceeds 18 dB across all channels, ensuring negligible inter-channel crosstalk and reliable first-layer filtering. This two-metalens configuration is a practical compromise to minimize experimental errors and obtain robust classification results, as noted in the main text.

S6. Phase Response of Rectangle Nanopillars

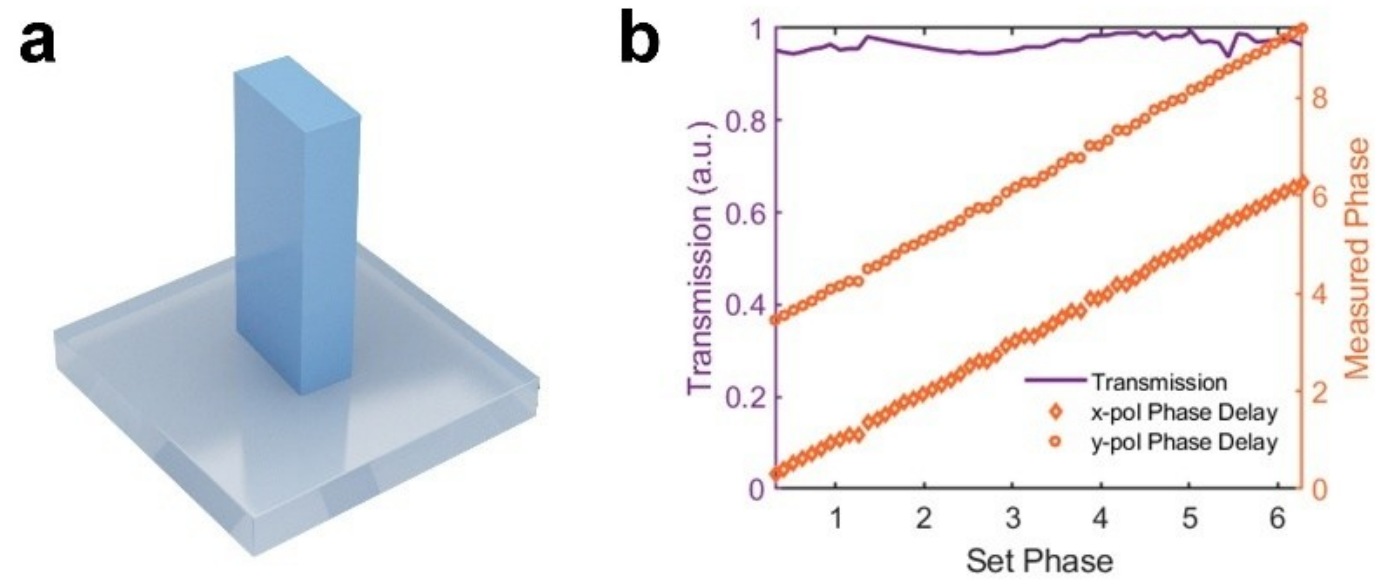

Figure S6. Phase Response of rectangle Nanopillars. (a) The structure of a single cell with a period of 520nm. (b) Screening curves are provided for phase and transmittance characteristics of individual nano units within MS.

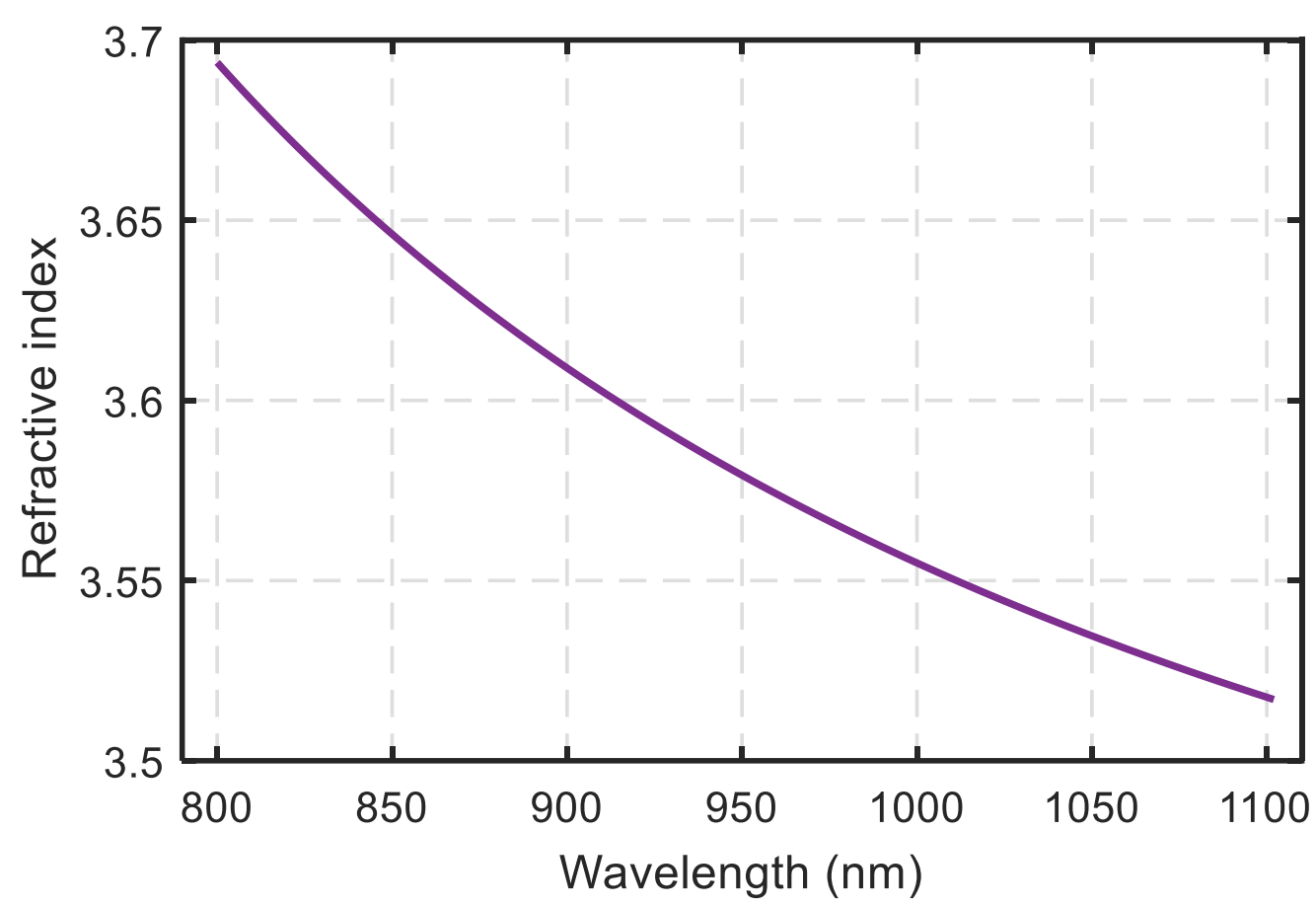

Figure S7. The measured refractive index (n, k) curves of the silicon.

The ±8 nm critical-dimension (CD) variation was determined by statistical analysis of scanning electron microscopy (SEM) images acquired from nine regions across the 2-mm aperture (3 × 3 grid, centre and edges). For each region, we measured 20 nanopillars (total 180

measurements). The mean CD was 220 nm, and the standard deviation was 8 nm, giving the reported ±8 nm variation.

S7. Amplitude prediction neural network (APNN)

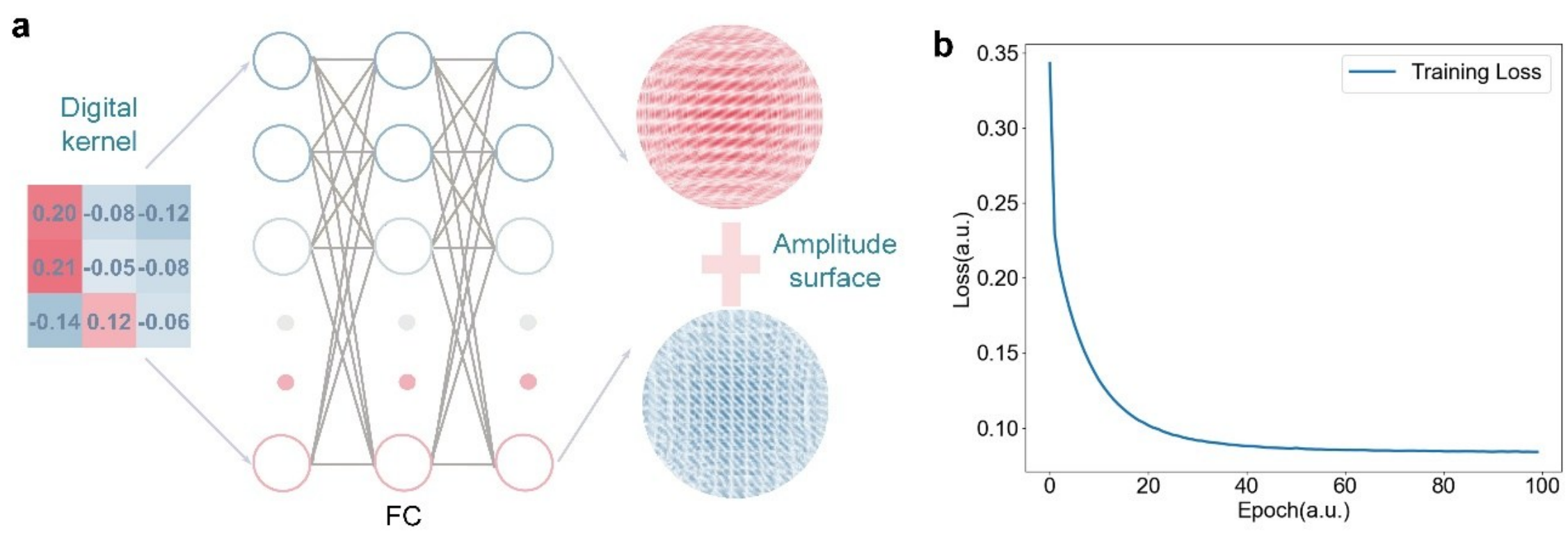

Figure S8. Amplitude prediction neural network (a) Diagram of APNN. (b) Convergence curve of loss function in neural network training.

APNN is a lightweight deep learning model designed for optoelectronic hybrid computing architectures, as shown in Fig. S8, which aims to quickly predict the optimal parameters of the dynamic amplitude modulation surface in a data-driven manner, thus replacing the traditional time-consuming gradient descent optimization process. The network is based on encoder-decoder structure, with the input as the intensity distribution of the target PSF and the output as the transmittance matrix (amplitude plane) of the liquid crystal layer. By training 4,000 sets of optimized data (input-output pairs) generated by gradient descent off-line, the APNN learns the nonlinear mapping from the target PSF to the amplitude plane and embedding physical constraints (such as conservation of energy, phase-amplitude coupling) into the loss function to ensure optical realizability of the predicted results. The amplitude surface iteration formula based on APNN is

$$T\big(V(x,y)\big)^{(t+1)} = T\big(V(x,y)\big)^{(t)} - \eta \cdot \frac{\partial \mathrm{CrossEntropy}\,(\hat{y}, \mathrm{y_{true}})}{\partial T\big(V(x,y)\big)} \tag{S8}$$

$T\big(V(x,y)\big)^{(t)}$ for the t iteration, the liquid crystal layer in space (x, y) transmittance (by voltage V (x y) control). η is the learning rate and $\mathrm{Cross\ Entropy}\,(\hat{y}, \mathrm{y_{true}})$ is the cross-entropy loss. Experiments show that APNN can generate a high-fidelity amplitude distribution in only 0.8ms, which is more than 2,800 times faster than traditional optimization methods, while maintaining classification task accuracy comparable to that of pure digital networks[6].

Number of pixels	1920*1080	Pixel
Pixel pitch	8	μm
Effective area size	15.36*8.64	mm^2
Reflectance	＞75	%
Fill factor	95	%
Input Frame Rate	60	Hz

Table S2. Specifications of LC-SLM.

We measured crosstalk both in simulation and experiment, defining it as the intensity leakage into neighboring spots when only one LC region is modulated. Simulation gave a maximum total crosstalk of -21 dB (average -24 dB). Experimental measurement yielded a maximum crosstalk

of -18.1 dB (i.e., < 1.6 % intensity leakage), with nearest-neighbor average of -19.2 dB. These values are well below the tolerance for accurate convolution.

S8. Optical Characterization Setup for Point Spread Function

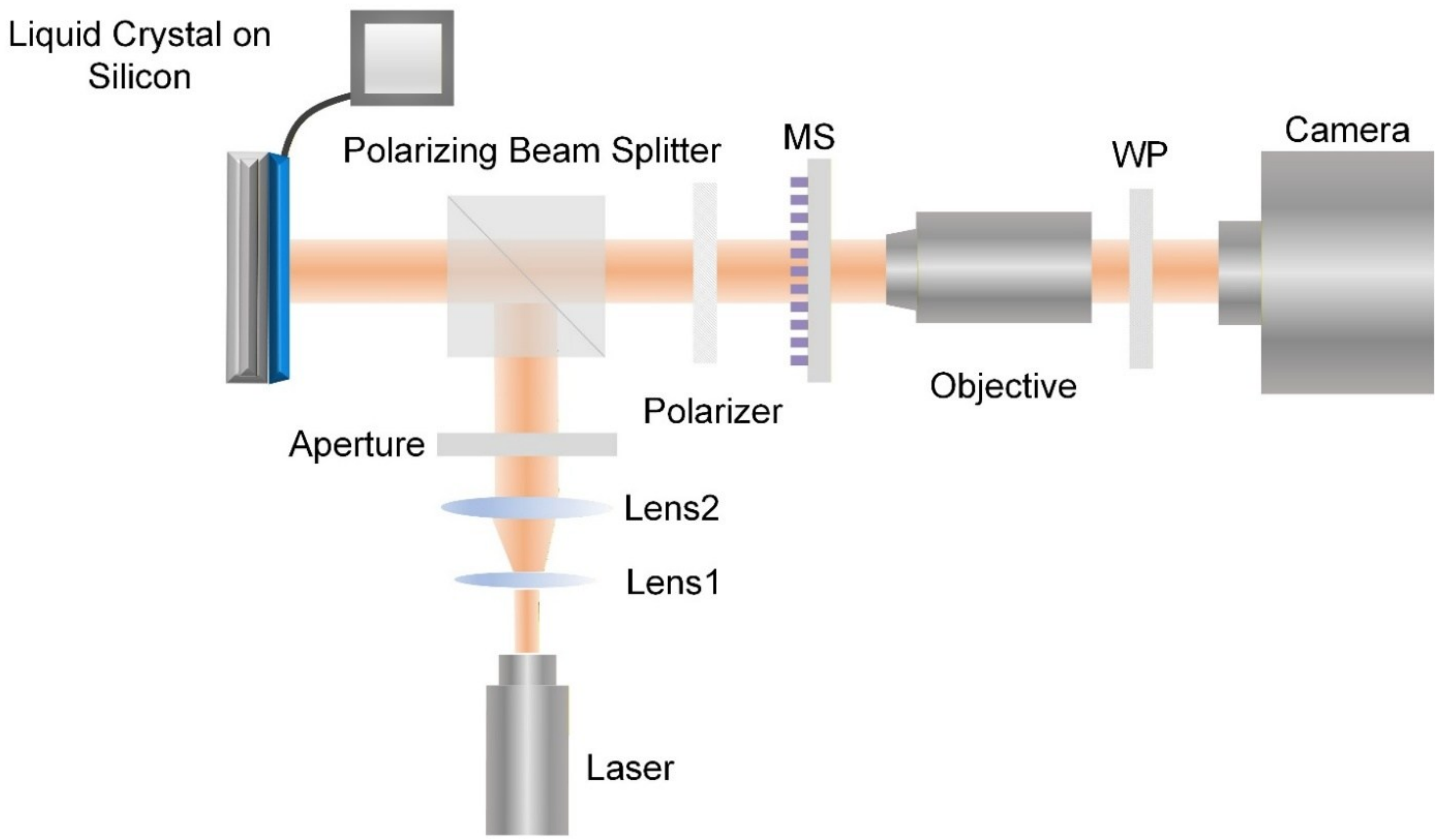

Figure S9. Schematic of the Characterization Setup for Point Spread Function.

For point-extended function characterization, a custom optical device was assembled. An objective lens was used in this setup. The illumination source is an infrared laser whose output is collimated through a lens. After PBS beam splitting to LC-SLM, the image and amplitude plane are loaded. After MS focused imaging and objective magnification, the point spread function map is captured by the photodetector.

S9. Experiment on wide-band PSF

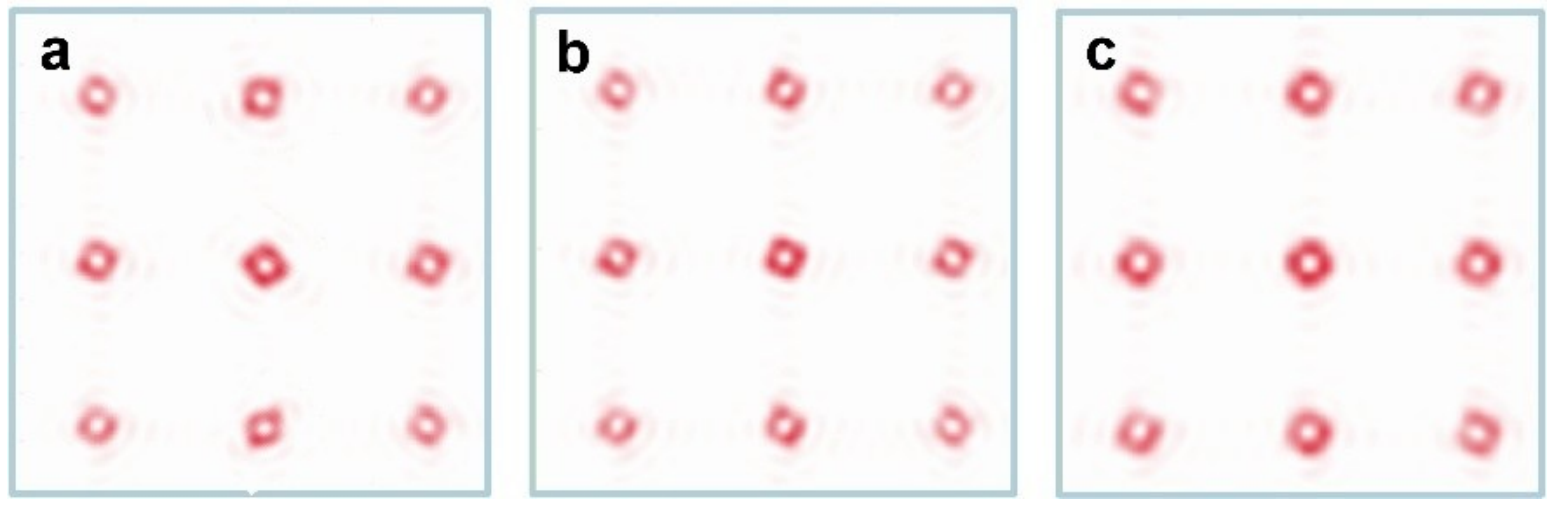

Figure S10. The intensity distribution of the PSF of the imager obtained through experimental measurement at incident wavelengths of (a)850nm, (b)950nm, and (c)1050nm.

The fabricated metasurface exhibits a measured diffraction efficiency of 62.4 % (into the 3×3 focal array) and a total transmittance of 87.3 % at 950 nm, corresponding to an insertion loss of 4.2 dB for the complete front end (metasurface + LC-SLM + polarizers). The polarization extinction ratio, measured for each of the four channels, averages 18.5 dB. The LC-SLM (PLUTO-2.1-NIR-015, 1920×1080 pixels, 8.0 μm pitch) operates at 60 Hz with a rise/fall time of 24/57 ms. With the LC frame rate and detector integration time, the total latency is approximately 160 ms, dominated by the modulator switching time. The power consumption of the optical front end (LC-SLM, CMOS, and illumination) is measured to be approximately 25.5 W, compared with approximately 170 W for the digital GPU backend. The detector used for classification is a polarization-sensitive camera (DZK 33UX250, 3.45 μm pixel pitch).

S10. The weight distribution of the three experiments

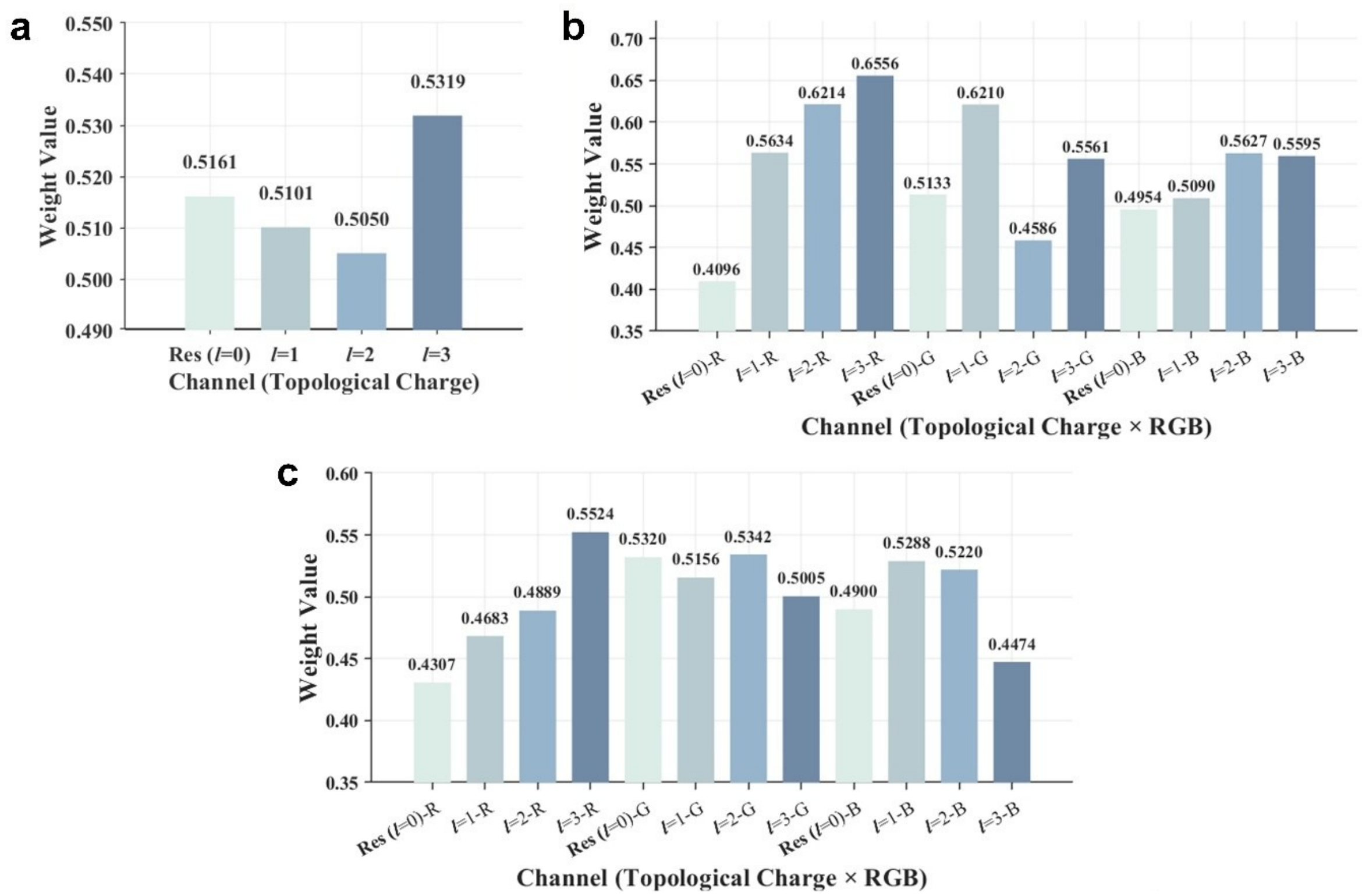

Figure S11. Learned channel weights across three datasets. Normalized learned channel weights for polarization channels *l*=0,1,2,3 on (a) Fashion-MNIST, (b) CIFAR-10, and (c) SIPaKMeD. The *l*=0 channel consistently exhibits a center-weighted residual distribution, while higher-order channels develop dataset-specific spatial structures.

Dataset	Training set	Test set	Batch size	Optimizer & Learning rate	Random seed
Fashion-MNIST	60 000	10 000	128	Adam, η=1×10^{-3}	42
CIFAR-10	50 000	10 000	64	Adam, η=1×10^{-3}	42
SIPaKMeD	2 429	810	32	Adam, η=1×10^{-3}	42

Table S3. Summary of dataset splits and training hyper-parameters used in classification experiments.

S11. Optical Characterization Setup for Object Classification

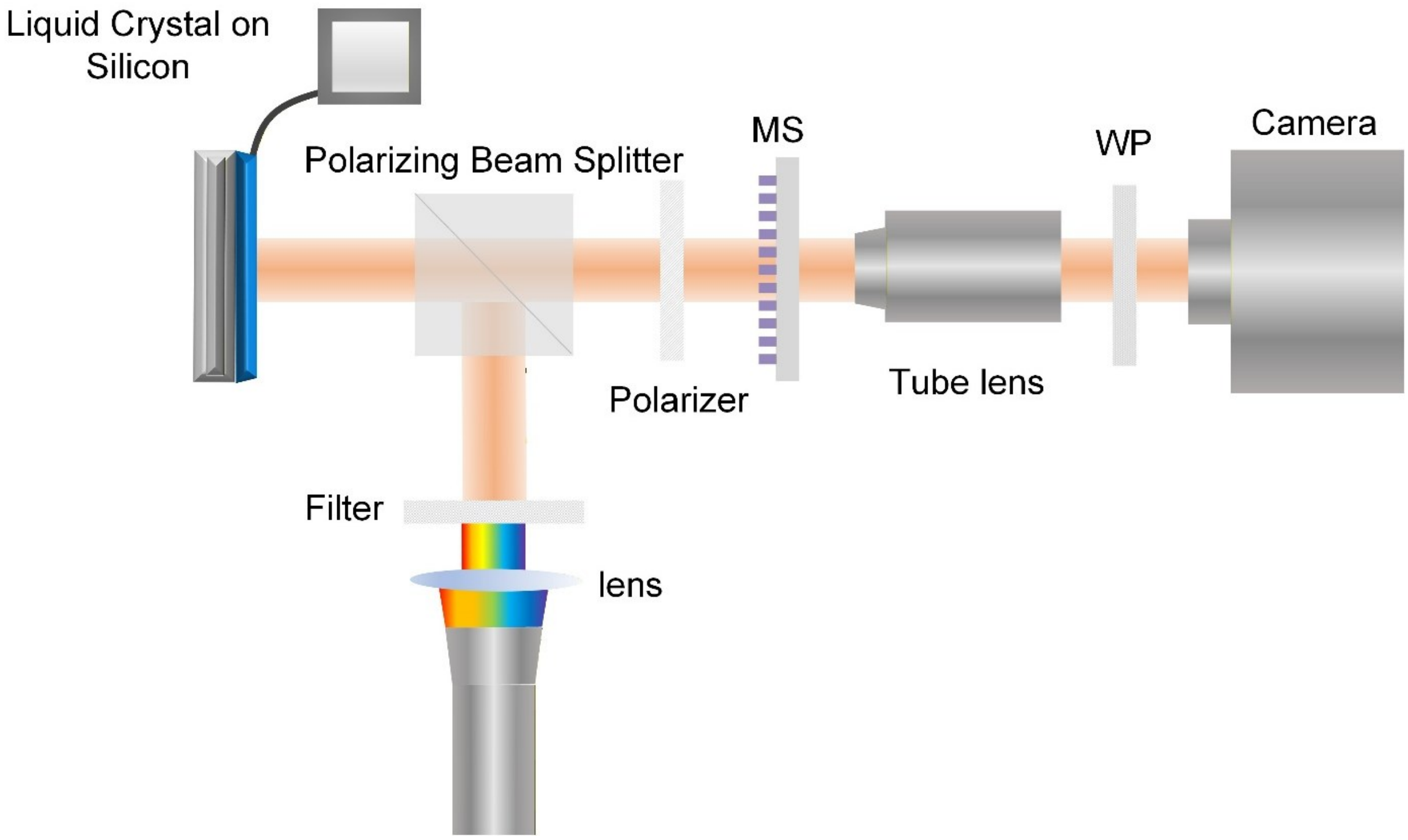

Figure S12. Schematic of the Characterization Setup for Object Classification Tasks.

In order to generate optical images, a machined LCOS was used. The LCOS can modulate the amplitude of light. The lighting system consists of an incoherent light source and a bandpass filter to select a specific wavelength range. A spherical lens acted as a concentrator, appropriately focusing the light for the experiment. Then, the optical convolution of the feature mapping was performed by using the MS. These feature maps were captured by a photodetector. To help record the scene, a 10X objective lens (DHC GCO-2105) was placed in front of the camera. This setup allows for precise and accurate data acquisition related to the optical convolution process.

We clarify that all reported "optical" accuracies are experimentally measured from captured

optical frames. For each dataset, we physically displayed the test images on the LCOS, passed them through the meta-imager, captured the optically convolved feature maps with the CMOS camera, and fed these measured feature maps into the lightweight digital classifier. The digital convolution baselines are purely numerical simulations. The simulated PSF (Fig. 4c) is used only for design validation and is explicitly labelled as such.

S12. Feature mapping of Fashion-MNIST dataset

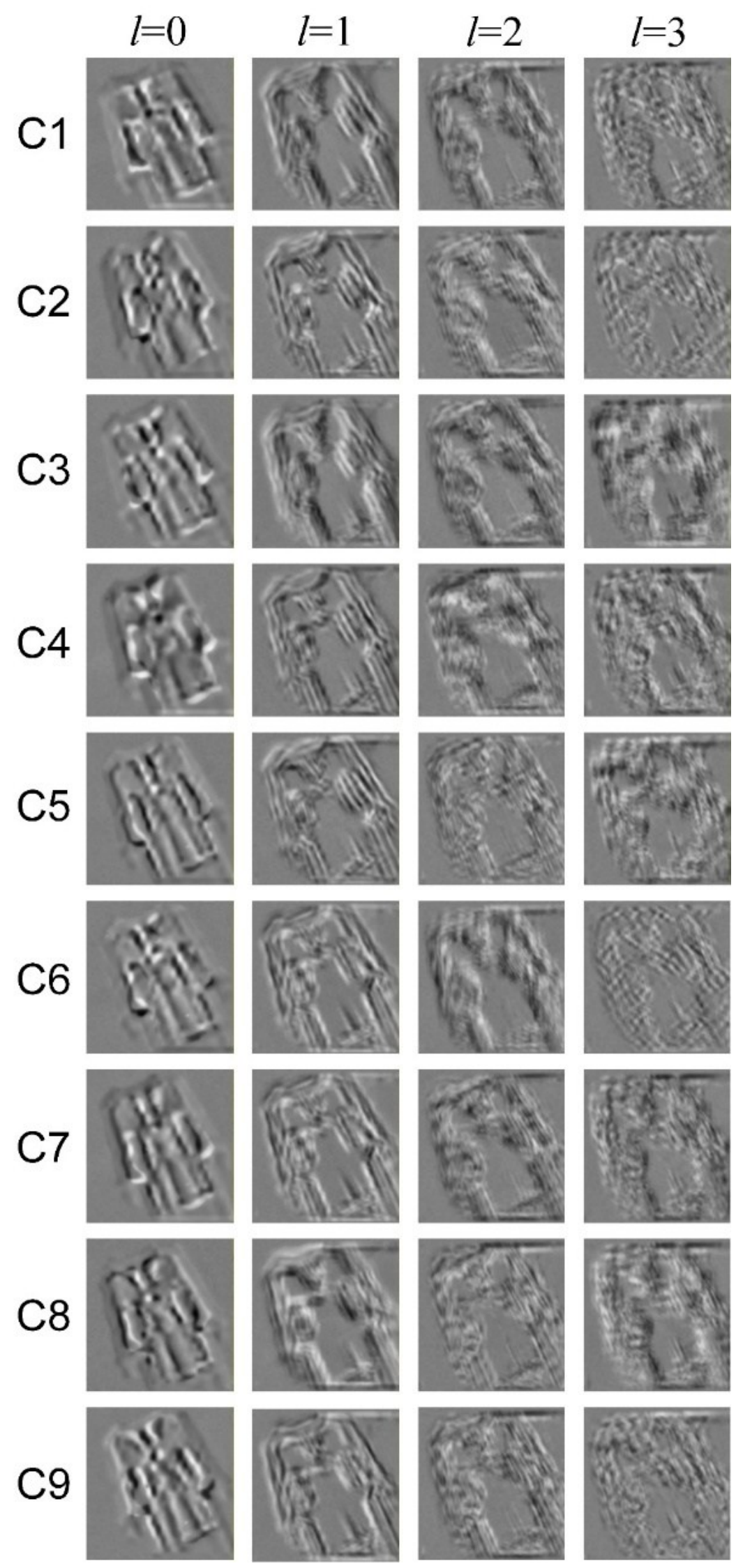

Figure S13. Feature mapping of Fashion-MNIST dataset.

S13. Feature mapping of CIFAR-10 dataset

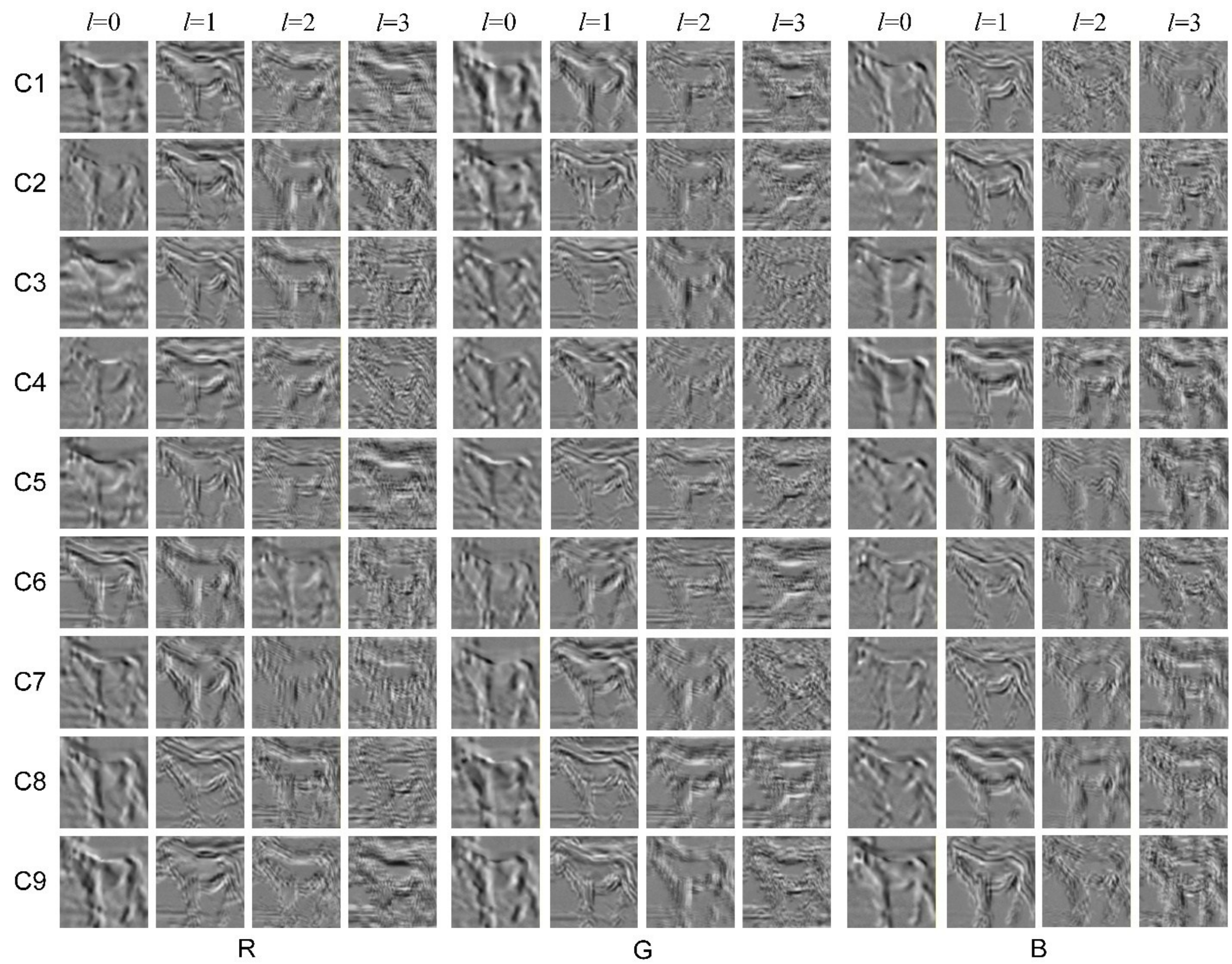

Figure S14. Feature mapping of CIFAR-10 dataset.

S14. Feature mapping of SIPaKMeD dataset

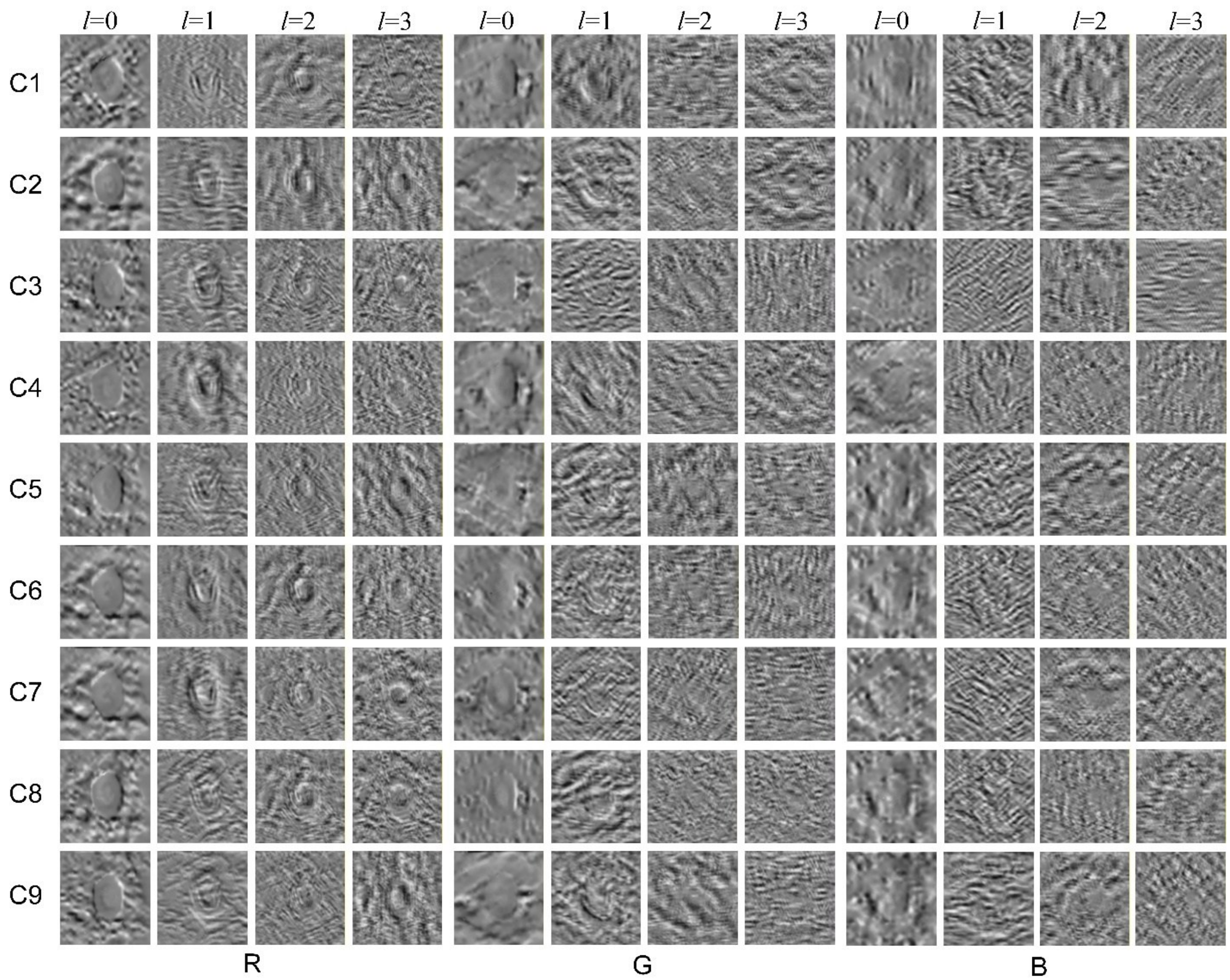

Figure S15. Feature mapping of SIPaKMeD dataset.

S15. Confusion matrices for three classification architectures across benchmark datasets

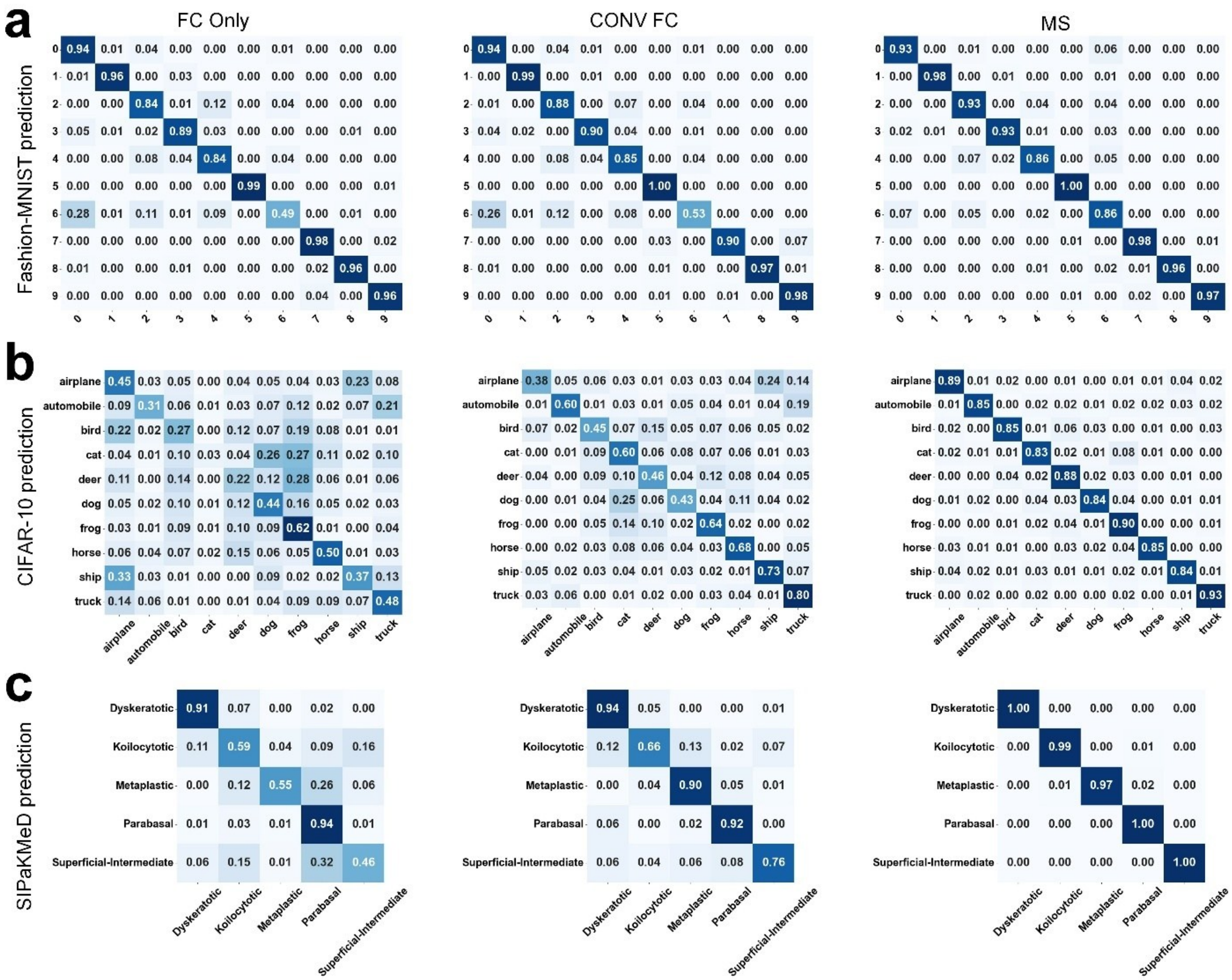

Figure S16. Confusion matrices for three classification architectures across benchmark datasets. Accuracy comparison of fully connected (FC), single-layer digital convolution (Conv), and optical convolution experiment on (a) Fashion-MNIST, (b) CIFAR-10, and (c) SIPaKMeD. The optical front end achieves the highest accuracy across all datasets, with the most pronounced gains on CIFAR-10 and SIPaKMeD.

Supplementary Figure 16 presents the confusion matrices and corresponding accuracies for three processing architectures evaluated on the three benchmark datasets. On Fashion-MNIST, the optical convolution attains 93.9%, modestly outperforming both the FC baseline (88.4%) and single-layer digital convolution (89.4%). For CIFAR-10, optical preprocessing raises accuracy from 36.9% (FC) and 57.5% (CONV FC) to 86.6%, demonstrating the critical role of multi-layer optical feature extraction for full-color natural imagery. On SIPaKMeD, the optical approach reaches 99.3%, substantially exceeding the FC (69.1%) and digital convolution (83.6%) baselines. These results confirm that embedding two effective convolutional layers within the meta-imager consistently enhances classification fidelity, particularly for complex, high-resolution tasks.

Dataset	FC-Only (simulated only)	1-layer digital conv (simulated only)	2-layer digital conv (simulated only)	This work (simulated only)	This work (experiment)
Fashion-MNIST	88.4%	89.4%	94.3%	94.1%	93.9%
CIFAR-10	36.9%	57.5%	87.5%	87.2%	86.6%
SIPaKMeD	69.1%	83.6%	99.6%	99.4%	99.3%

Table 4. Summary of classification accuracies (%) across all benchmark datasets and processing architectures.

We acknowledge that several figures in the original manuscript lacked error bars. We have now performed three independent measurement runs for all optical classification experiments (each run comprises a complete test-set inference with independent LC-SLM calibration and re-alignment).

Fig	Error bars (± σ)
Fig. 1b	±0.7%
Fig. 1c	±0.6%
Fig. 4e	±0.5%
Fig. 5g	±0.8%
Fig. 5h	±1%

Table S5. The following figures have been updated with error bars.

Wavelength (nm)	Accuracy (mean ± σ)
850	99.3% ± 0.5%
900	99.2% ± 0.4%
950	99.5% ± 0.3%
1000	99.0% ± 0.5%
1050	99.1% ± 0.6%

Table S6. The revised Fig. 1c includes error bars.

Condition	Optical system (experimentally measured)	Digital 2-layer baseline (simulated)
Unrotated Fashion-MNIST	94.0 % ± 0.7 %	94.3 % ± 0.2 %
Rotated Fashion-MNIST	93.9 % ± 0.5 %	91.7 % ± 0.4 %

Table S7. Classification accuracy comparison on Fashion-MNIST under rotated and unrotated conditions.

Crucially, the digital baseline for rotation robustness was trained without any rotation augmentation, so the 2.6 percentage-point drop (94.3% → 91.7%) reflects the digital network's sensitivity to rotation. In contrast, the optical front end drops only 0.2 percentage points (94.1% → 93.9%), demonstrating that the robustness indeed originates from the Bessel–vortex basis rather than from training. We also clarify that the optical system itself was never trained on rotated images; the robustness is inherent to the optical kernel design.

S16. Prediction accuracy analysis for Fashion MNIST dataset, CIFAR-10, and SIPaKMeD.

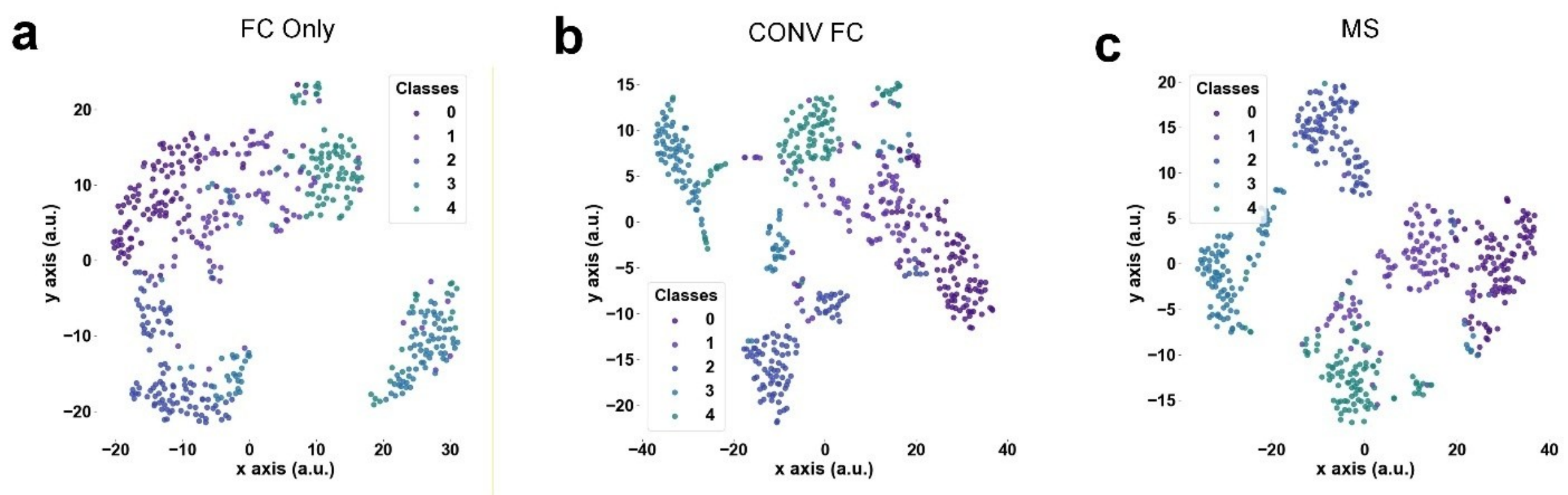

Figure S17. The t-SNE visualization of feature embeddings for SIPaKMeD. Two-dimensional t-SNE projections of feature representations from (a) fully connected network, (b) single-layer digital convolution, and (c) optical convolution via the meta-imager. Points are colored by the five diagnostic cell categories. Optical preprocessing yields the most distinct inter-class separation and compact intra-class clustering.

The t-SNE visualizations in Supplementary Figure S17 compare the feature-space organization produced by different processing pipelines on the SIPaKMeD dataset. Fully connected features (a) exhibit substantial class overlap. Single-layer digital convolution (b) improves separation but retains ambiguous boundaries. Optical convolution (c) achieves the clearest cluster separation and tightest intra-class grouping, consistent with the 99.6% classification accuracy reported in the main text. This confirms that the Bessel-vortex priors and tunable vortex array extract highly discriminative morphological features from high-resolution cytology images.

References

1. Y. LeCun, Y. Bengio, and G. Hinton, “Deep Learning,” *Nature 521*, no. 7553 (2015): 436–444, https://doi.org/10.1038/nature14539.

2. S. Furui, L. Deng, M. Gales, H. Ney, and K. Tokuda, “Fundamental Technologies in Modern Speech Recognition [From the Guest Editors],” *IEEE Signal Processing Magazine 29*, no. 6 (2012): 16–17, https://doi.org/10.1109/MSP.2012.2209906.

3. L. Liu, S. Lu, R. Zhong, et al., “Computing Systems for Autonomous Driving: State of the Art and Challenges,” *IEEE Internet of Things Journal 8*, no. 8 (2021): 6469–6486, https://doi.org/10.1109/JIOT.2020.3043716.

4. L. Mennel, J. Symonowicz, S. Wachter, D. K. Polyushkin, A. J. Molina-Mendoza, and T. Mueller, “Ultrafast Machine Vision with 2D Material Neural Network Image Sensors,” *Nature 579*, no. 7797 (2020): 62–66, https://doi.org/10.1038/s41586-020-2038-x.

5. A. Arbabi, Y. Horie, M. Bagheri, and A. Faraon, “Dielectric Metasurfaces for Complete Control of Phase and Polarization with Subwavelength Spatial Resolution and High Transmission,” *Nature Nanotechnology 10*, no. 11 (2015): 937–943, https://doi.org/10.1038/nnano.2015.186.

6. S. Li, X. Li, G. Wang, et al., “Multidimensional Manipulation of Photonic Spin Hall Effect with a Single-Layer Dielectric Metasurface,” *Advanced Optical Materials 7*, no. 5 (2019): 1801365, https://doi.org/10.1002/adom.201801365.

7. W. Shi, Z. Huang, H. Huang, et al., “LOEN: Lensless Opto-Electronic Neural Network

Empowered Machine Vision," *Light: Science & Applications 11*, no. 1 (2022): 121, https://doi.org/10.1038/s41377-022-00809-5.
